# Molecular imaging of HER2 expression in breast cancer patients using a novel peptide-based tracer ^99m^Tc-HP-Ark2: a pilot study

**DOI:** 10.1186/s12967-022-03865-y

**Published:** 2023-01-11

**Authors:** Jiyun Shi, Shuaifan Du, Rongxi Wang, Hannan Gao, Qi Luo, Guozhu Hou, Yidong Zhou, Zhaohui Zhu, Fan Wang

**Affiliations:** 1 Medical Isotopes Research Center and Department of Radiation Medicine, State Key Laboratory of Natural and Biomimetic Drugs, School of Basic Medical Sciences, International Cancer Institute, Peking University, Beijing, 100191 China; 2 Key Laboratory of Protein and Peptide Pharmaceuticals, CAS Center for Excellence in Biomacromolecules, Institute of Biophysics, Chinese Academy of Sciences, Beijing, 100101 China; 3 Department of Nuclear Medicine, State Key Laboratory of Complex Severe and Rare Diseases, Beijing Key Laboratory of Molecular Targeted Diagnosis and Therapy in Nuclear Medicine, Peking Union Medical College Hospital, Chinese Academy of Medical Sciences, Peking Union Medical College, Beijing, 100730 China; 4 Guangzhou Laboratory, Guangzhou, 510005 China; 5 Department of Breast Surgery, Peking Union Medical College Hospital, Beijing, 100730 China

**Keywords:** SPECT/CT, HER2, Breast cancer, ^99m^Tc, Trastuzumab

## Abstract

**Background:**

Due to the temporal and spatial heterogeneity of human epidermal growth factor receptor 2 (HER2) expression in breast tumors, immunohistochemistry (IHC) cannot accurately reflect the HER2 status in real time, which may cause misguided treatment decisions. HER2-specific imaging can noninvasively determine HER2 status in primary and metastatic tumors. In this study**,** HER2 expression in breast cancer patients was determined in vivo by SPECT/CT of ^99m^Tc-HP-Ark2, comparing with PET/CT of ^18^F-FDG lesion by lesion.

**Methods:**

A novel HER2-targeted peptide probe ^99m^Tc-HP-Ark2 was constructed. Biodistribution and nanoScan SPECT/CT imaging were performed in mice models. The correlation between the quantified tumor uptake and HER2 expression in tumor cells was analyzed. In the pilot clinical study, a total of 34 breast cancer patients (mean age ± SD: 49 ± 10 y) suspected of having breast cancer according to mammography or ultrasonography were recruited at Peking Union Medical College Hospital, and ^99m^Tc-HP-Ark2 SPECT/CT and ^18^F-FDG PET/CT were carried out with IHC and fluorescence in situ hybridization as validation.

**Results:**

Small animal SPECT/CT of ^99m^Tc-HP-Ark2 clearly identified tumors with different HER2 expression. The quantified tumor uptake and tumor HER2 expression showed a significant linear correlation (r = 0.932, P < 0.01). Among the 36 primary lesions in the 34 patients, when IHC (2 +) or IHC (3 +) was used as the positive evaluation criterion, ^99m^Tc-HP-Ark2 SPECT/CT imaging with a tumor-to-background ratio of 1.44 as the cutoff value reflected the HER2 status with sensitivity of 89.5% (17/19), specificity of 88.2% (15/17) and accuracy of 88.9% (32/36), while the ^18^F-FDG PET/CT showed sensitivity of 78.9% (15/19), specificity of 70.6% (12/17) and accuracy of 75.0% (27/36). In particular, 100% of IHC (3 +) tumors were all identified by ^99m^Tc-HP-Ark2 SPECT/CT imaging.

**Conclusion:**

^99m^Tc-HP-Ark2 SPECT/CT can provide a specific, noninvasive evaluation of HER2 expression in breast cancer, showing great potential to guide HER2-targeted therapies in clinical practice.

ClinicalTrials.gov Trial registration: NCT04267900. Registered 11th February 2020. Retrospectively registered, https://www.clinicaltrials.gov/ct2/results?pg=1&load=cart&id=NCT04267900.

**Supplementary Information:**

The online version contains supplementary material available at 10.1186/s12967-022-03865-y.

## Background

Human epidermal growth factor receptor 2-positive (HER2^+^) breast cancer accounts for 20–30% of invasive breast cancers, and HER2 expression is associated with poor prognosis [1, 2]. HER2^+^ patients can benefit from HER2-targeted treatment [3, 4]. Trastuzumab (Herceptin^®^, Genentech, Inc.), as an anti-HER2 monoclonal antibody, is currently the first-line drug for HER2^+^ breast cancer, as it significantly improves the chances of cure of early HER2^+^ breast cancer and reduces the risks of recurrence and death [5, 6]. However, the approximately 30% response rate and varying degrees of drug resistance make the accurate diagnosis of HER2 expression essential [7]. In addition, the downregulation of HER2 expression is reported as an indicator of trastuzumab’s antitumor effect [8, 9]. Therefore, HER2 expression assessment is important not only before but also during treatment. In current clinical practice, biopsy-based immunohistochemistry (IHC) and fluorescence in situ hybridization (FISH) are recognized as the gold standards for detecting HER2 status [10]. However, metastases are often found in breast cancer patients, and it is difficult to test the HER2 status of bone metastases by biopsy. It is also difficult for biopsy to identify heterogeneity and discordance of HER2 status between metastases and primary lesions. The temporal and spatial heterogeneity of HER2 expression in breast tumors can lead to inaccurate assessments and further mislead oncologists in choosing the therapeutic regimen [11]. The shortcomings of invasive biopsy analysis have promoted the development of HER2-targeted molecular imaging [12].

Molecular imaging probes targeting HER2 have been extensively studied [13–15]. In some clinical studies, the therapeutic antibodies trastuzumab and pertuzumab have been radiolabeled to guide the administration of targeted therapy. However, antibody-based imaging usually needs to wait 2–4 days after administration, which is not convenient to guide treatment decisions in time and expose patients to longer radiation exposure [16–19]. In addition, radiolabeled antibodies usually have a high liver background uptake, which may lead to poor visualization of liver metastases [20]. Therefore, many small molecule substitutes targeting HER2 have emerged, including antibody fragment [21], nanobody [22], affibody [23] and peptide [24], which have faster blood circulation and better tissue permeability and are more suitable for the development of imaging probes than antibodies, and some of them have been developed as cancer diagnostic probes for clinical trials.

Peptides have many favorable characteristics suitable for the development of imaging agents [24, 25]. In addition to high tissue permeability and fast blood clearance, they are also easy to synthesize and formulate kits, which is more conducive to clinical translation and promotion. However, at present, almost all HER2-targeted peptide probes are in preclinical studies, and clinical translational studies are still scarce. Previously, we have developed two probes, ^99m^Tc-H6F and ^99m^Tc-H10F, based on two HER2-targeting peptides that bind to HER2 at different binding sites with trastuzumab (extracellular II *vs.* extracellular IV) and thus hold the potential to monitor efficacy during trastuzumab therapy. However, ^99m^Tc-H6F has poor water solubility and high lipophilicity, resulting in high gallbladder uptake, which is not conducive to clinical use [26]. ^99m^Tc-H10F has good water solubility, and its ability to monitor treatment efficacy has been verified in animal models. However, its rapid clearance in vivo and relatively low tumor uptake also limit its further clinical application [27]. In this study, we developed an improved HER2-targeting molecular probe ^99m^Tc-HP-Ark2 based on H10F peptide through D-shaped amino acids, sequence reversal, dimerization, 8-carbon aliphatic chain and PEG_4_ chain modification, etc. Compared with the previous probe ^99m^Tc-H10F [27], ^99m^Tc-HP-Ark2 had enhanced HER2 targeting capability and improved pharmacokinetic properties, thereby increasing its efficacy for the clinical detection of HER2 expression. A pilot prospective clinical study of ^99m^Tc-HP-Ark2 SPECT/CT (single photon emission computed tomography/computed tomography) was performed in breast cancer patients and compared with ^18^F-FDG PET/CT (positron emission tomography/computed tomography) lesion by lesion.

## Results

### Chemistry

**Fig. 1:**
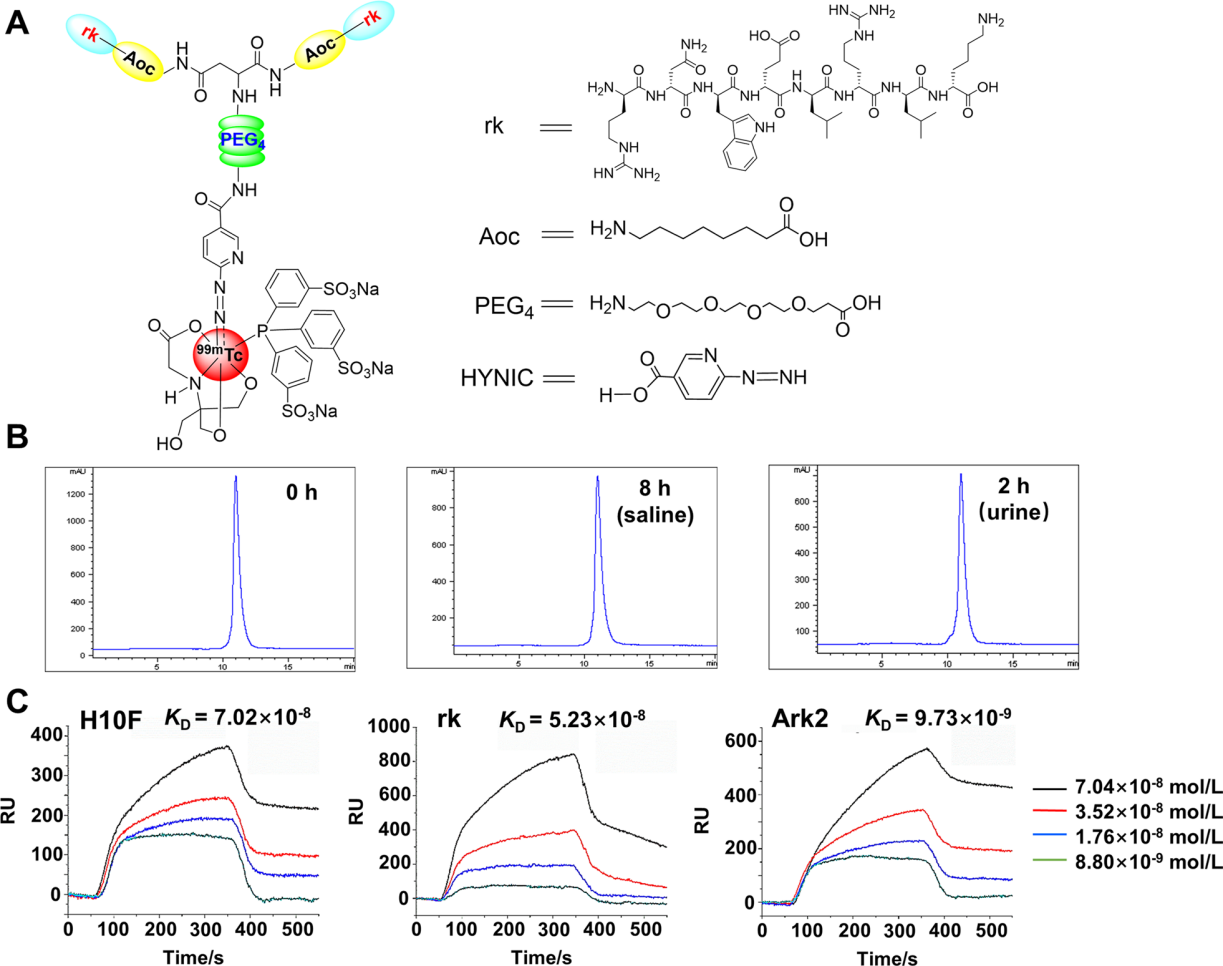
Chemical structure, stability and binding affinity. **A** Structure of ^99m^Tc-HP-Ark2. **B** In vitro stability and in vivo metabolic stability of ^99m^Tc-HP-Ark2. **C** Surface plasmon resonance results of H10F (70.2 nM), rk (52.3 nM) and Ark2 (9.7 nM) toward HER2

Firstly, the l-amino acid sequence (KLRLEWNR, named H10F) was replaced with a reversed D-amino acid sequence (sequence: _D_RNWELRLK, termed rk). Subsequently, 8-aminooctanoic acid (Aoc) as a linker was conjugated to rk (termed Ark), and then the Ark peptide was dimerized (termed Ark2), and further conjugated with HYNIC chelator via PEG_4_ liner (termed HP-Ark2) for ^99m^Tc-radiolabeling (termed ^99m^Tc-HP-Ark2). The structures of rk and ^99m^Tc-HP-Ark2 are shown in Fig. 1A. The synthetic route of HP-Ark2 is shown in Additional file 1: Figure S1. The final product HP-Ark2 was obtained with a purity of > 98%, which was identified by mass spectrometry (m/z, 3172.5 for [M + H]^+^) (Additional file 1: Figure S2). The radiotracer ^99m^Tc-HP-Ark2 was prepared at a high labeling yield (> 98%) with a specific activity of > 14.8 MBq/μg and showed excellent in vitro and in vivo stability (Fig. 1B), while the parental L-peptide monomer tracer ^99m^Tc-H10F showed poor metabolic stability (Additional file 1: Figure S3).

### In vitro biological evaluation

As the D-enantiomer of the L-peptide H10F, the rk peptide maintains the similar spatial configuration of H10F, and it should retain similar binding properties to HER2. The dimerized peptide Ark2 was supposed to further enhance its binding affinity to HER2. To verify the difference in HER2-binding capability between H10F, rk and Ark2 peptides, the dissociation constants (KD) of the peptide ligands to HER2 protein were determined by surface plasmon resonance (SPR). They were calculated from the kinetic constants obtained by curve-fitting of the association and dissociation rates to real-time binding and releasing data. The binding affinity of the retro-inverso D-peptide rk (52 nM) to HER2 protein was comparable to that of H10F (70 nM). The dimer peptide Ark2 (10 nM) showed significantly enhanced binding affinity (Fig. 1C). Furthermore, the rk peptide bound specifically to HER2 but not to other proteins in the EGFR family (Additional file 1: Figure S4).

**Fig. 2:**
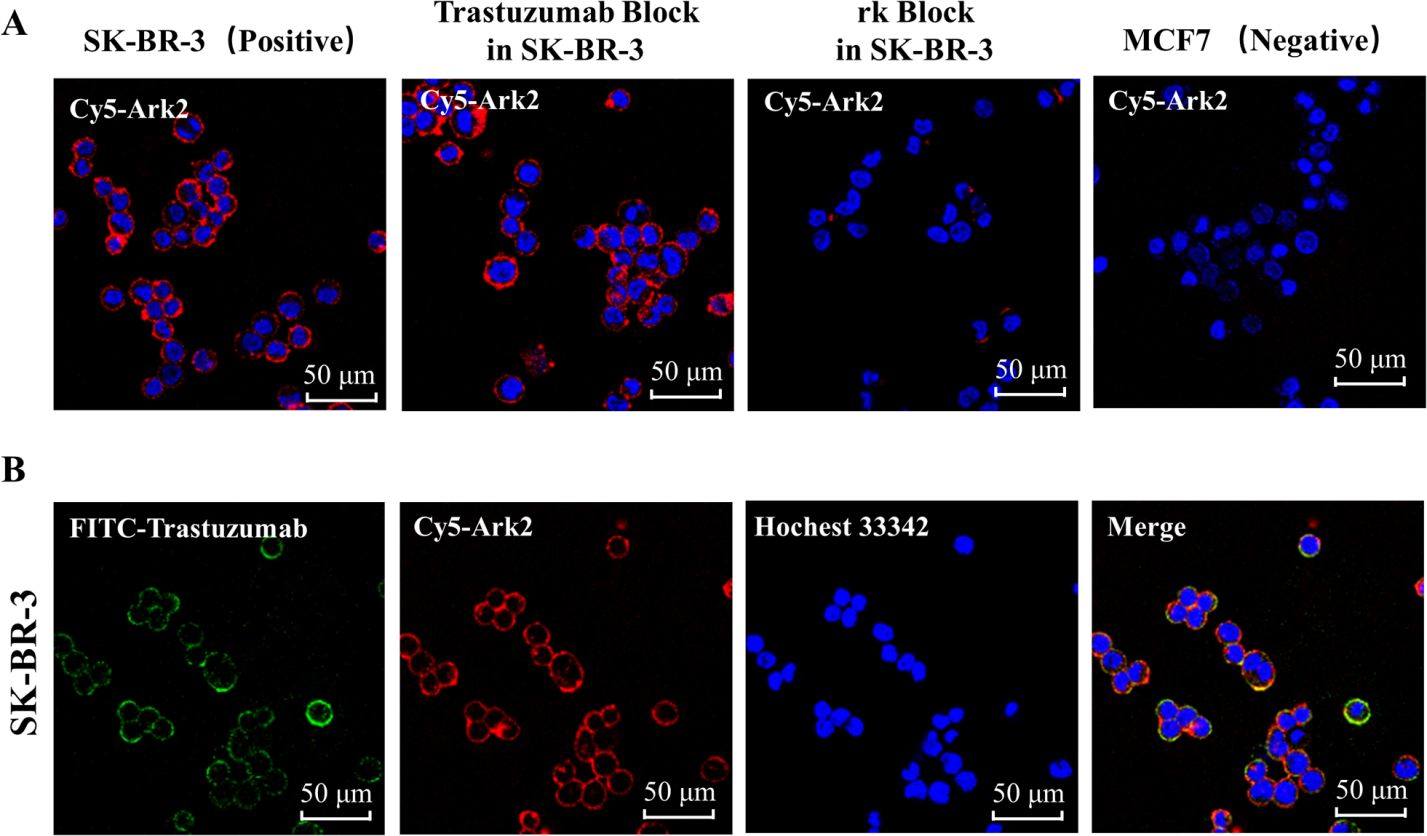
Cy5-Ark2 binding to cell lines on confocal microscopy. **A** Confocal images of Cy5-Ark2 binding to SK-BR-3 (HER2-positive) and MCF7 (HER2-negative) cells with/without trastuzumab or rk peptide blocking. **B** Colocalized staining of SK-BR-3 cells with FITC-trastuzumab and Cy5-Ark2

To further demonstrate the specific binding of Ark2 to HER2, HER2-positive SK-BR-3 cells and HER2-negative MCF7 cells were incubated with Cy5-Ark2, and the cell-associated fluorescence was visualized by confocal microscopy. As shown in Fig. 2A, intense fluorescence signals were found on the cell membranes of HER2-positive SK-BR-3 cells but barely found on HER2-negative MCF7 cells. Moreover, the intense fluorescence signals on the cell membrane of SK-BR-3 could be significantly blocked by 200-fold excess of cold rk peptide, demonstrating that the binding of Cy5-Ark2 to SK-BR-3 was specifically mediated by HER2 receptor. We further co-located the fluorescence signals of Cy5-Ark2 and fluorescence-coupled anti-HER2 antibody (FITC-trastuzumab) on the surface of SK-BR-3 cells, and the results showed that their signals had good overlap, which verified that they bound to the same receptor (Fig. 2B). However, excessive trastuzumab did not block the fluorescence signal of Cy5-Ark2 (Fig. 2A), indicating that Ark2 and trastuzumab bound to different epitopes of the HER2 protein without interaction.

### In vivo behavior of ^99m^Tc-HP-Ark2

**Fig. 3:**
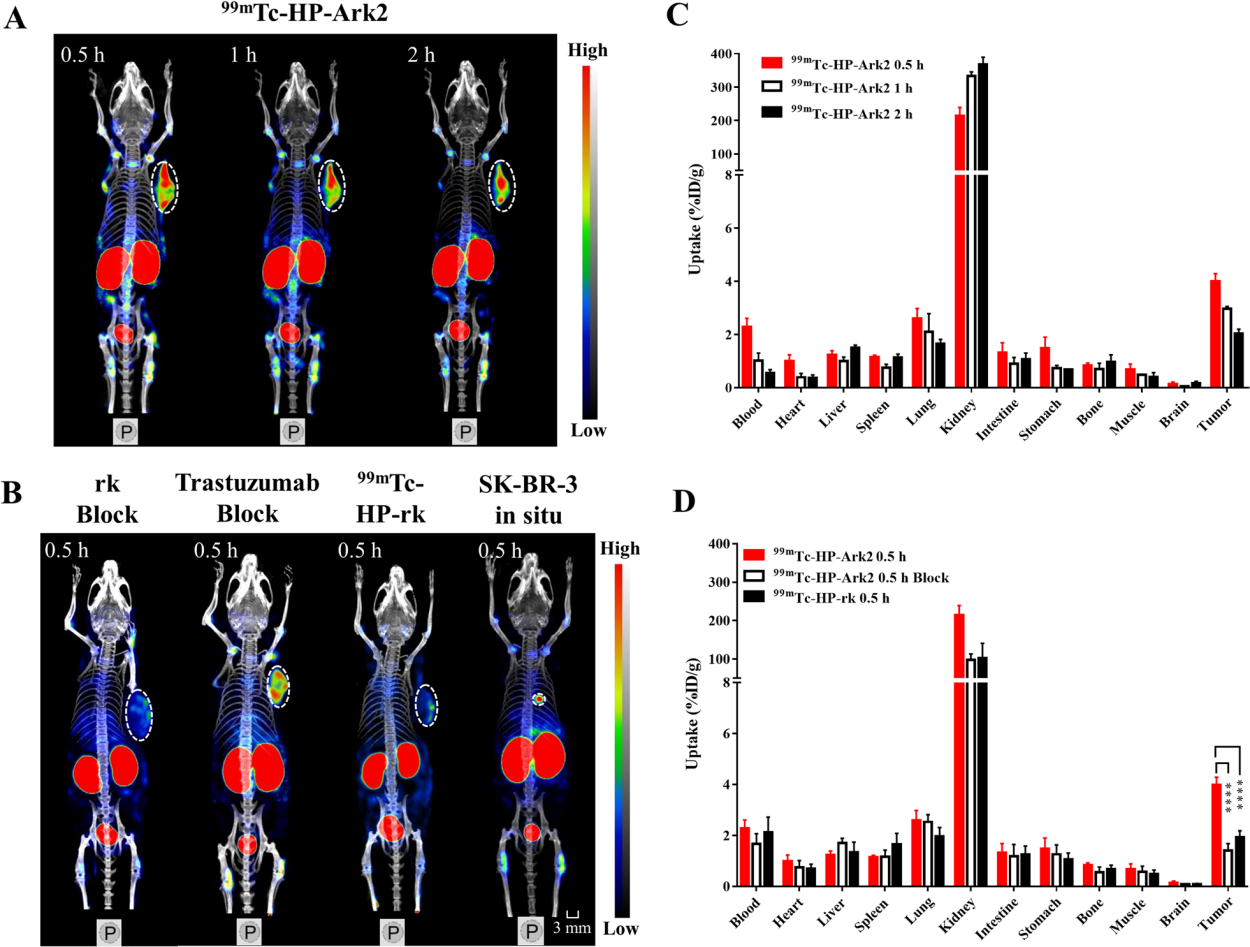
In vivo behavior of ^99m^Tc-HP-Ark2. **A** SPECT/CT images of ^99m^Tc-HP-Ark2 in the SK-BR-3 tumor model at 0.5, 1 and 2 h p.i. **B** SPECT/CT images of ^99m^Tc-HP-Ark2 (in situ tumor and blocking study) and ^99m^Tc-HP-rk in the SK-BR-3 tumor model at 0.5 p.i. **C** Biodistribution of ^99m^Tc-HP-Ark2 in the SK-BR-3 tumor model at 0.5, 1 and 2 h p.i. **D** Biodistribution of ^99m^Tc-HP-Ark2 with/without a blocking dose of cold rk peptide and ^99m^Tc-HP-rk in the SK-BR-3 tumor model at 0.5 h p.i

The in vivo tumor targeting capability of ^99m^Tc-HP-Ark2 was determined in the SK-BR-3 tumor model by nanoScan SPECT/CT imaging. The SK-BR-3 tumors could be clearly visualized at 0.5, 1 and 2 h postinjection (p.i.) (Fig. 3A), even when the tumor was as small as ~ 20 mm^3^, regardless of whether it was subcutaneous or an in situ model (Additional file 1: Figure S5 & Fig. 3B). Coinjection of excessive cold rk peptide significantly blocked tumor uptake, but excessive cold trastuzumab did not block tumor uptake (Fig. 3B), suggesting that the accumulation of ^99m^Tc-HP-Ark2 in HER2-positive tumors was specifically receptor-mediated and had no cross-interference by trastuzumab. The imaging intensity of ^99m^Tc-HP-Ark2 in tumors was stronger than that of ^99m^Tc-HP-rk (Fig. 3A, B).

The biodistribution results were consistent with the imaging results. ^99m^Tc-HP-Ark2 showed higher tumor uptake than uptake in other organs (except for kidneys) at all three studied time points, 0.5, 1 and 2 h p.i. (Fig. 3C), resulting in a better contrast of tumor-to-background. ^99m^Tc-HP-Ark2 showed notably enhanced tumor uptake (3.99 ± 0.15%ID/g) at 0.5 h p.i. compared to ^99m^Tc-HP-rk (1.94 ± 0.12 %ID/g, P < 0.0001, n = 4), with no significant difference in other organs except the kidneys. In the blocking group, the tumor uptake was significantly blocked by coinjection of excess cold rk peptide (1.42 ± 0.15 %ID/g, P < 0.0001, n = 4), indicating the specific targeting of the probe to HER2-positive tumors (Fig. 3D). In the control groups, the tumor uptake of ^99m^Tc-HP-Ark2 in the HER2-negative MCF7 (1.44 ± 0.08 %ID/g, P < 0.0001, n = 4) and MDA-MB-468 models (1.08 ± 0.06 %ID/g, P < 0.0001, n = 4) was significantly lower than that in the HER2-positive SK-BR-3 model, also indicating the specific targeting of the probe to HER2-positive tumors (Additional file 1: Figure S6). The specificity of Ark2 for HER2 was further verified by immunofluorescence staining of ex vivo tumor tissues. The results showed that Cy5-Ark2 could colocalize with trastuzumab in HER2-positive SK-BR-3 and MCF7-HER2 tumor tissues, while no obvious staining signals were detected in HER2-negative MCF7 tumor tissues (Additional file 1: Figure S7).

### Correlation between tumor uptake and HER2 expression

**Fig. 4:**
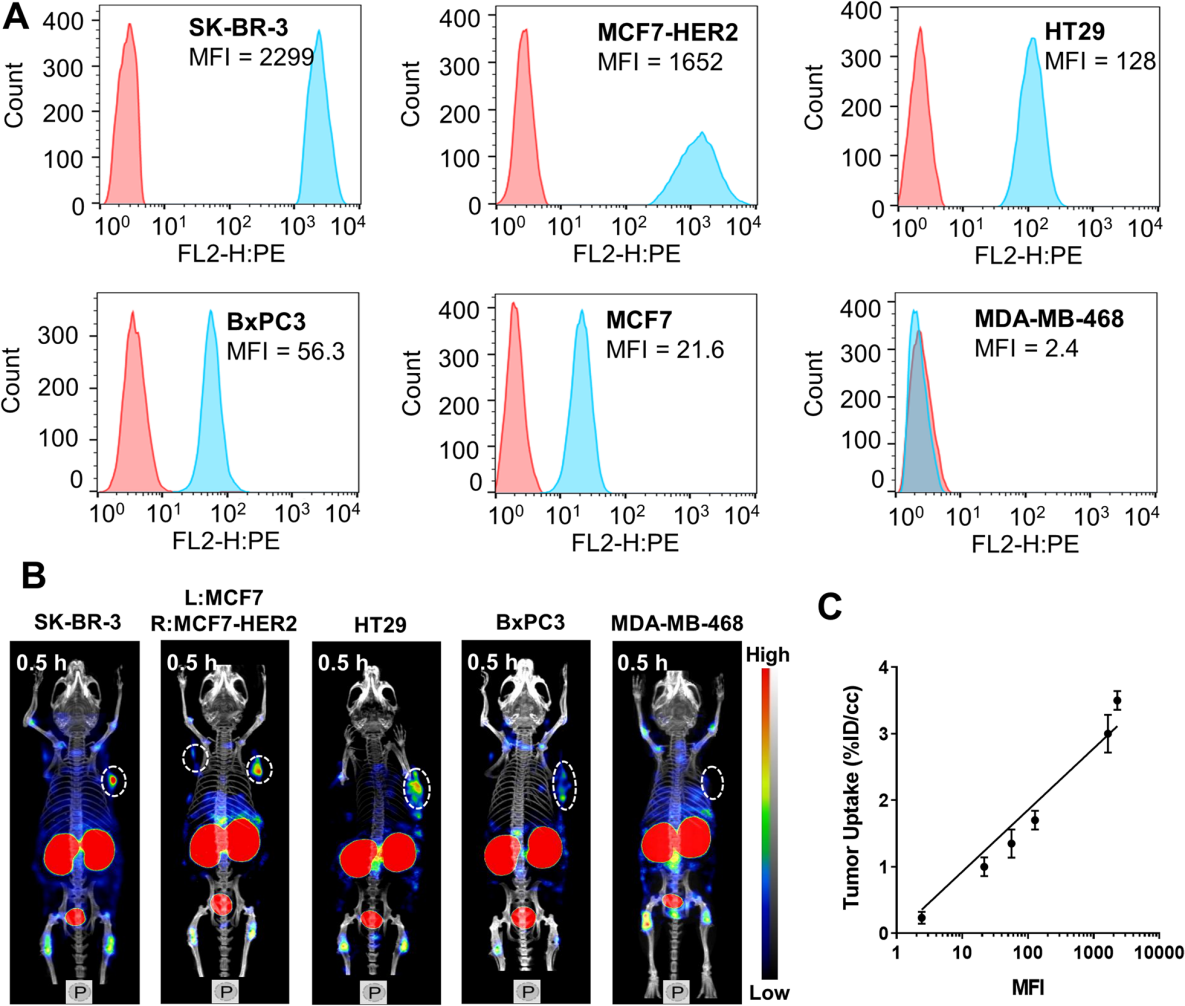
Correlation between tumor uptake and HER2 expression. **A** Representative flow cytometry histograms of different tumor cell lines. **B** NanoScan SPECT/CT images of ^99m^Tc-HP-Ark2 in different tumor models. **C** Correlation between tumor uptake and HER2 expression (MFI). MFI = mean fluorescence intensity

HER2 expression was measured in the selected tumor cell lines by flow cytometry (Fig. 4A). The lines were ranked from high to low HER2 expression as SK-BR-3 cells (mean fluorescence intensity, MFI: 2299), MCF7-HER2 cells (MFI: 1652), HT29 cells (MFI: 128), BxPC3 cells (MFI: 56.3), MCF7 cells (MFI: 21.6) and MDA-MB-468 (MFI: 2.42). SPECT/CT imaging was performed in the corresponding tumor models (Fig. 4B), and their uptake was quantified for correlation analysis. The quantified ^99m^Tc-HP-Ark2 tumor uptake and HER2 expression level had a linear correlation with r = 0.932 (P < 0.01) (Fig. 4C).

### Pilot clinical application of ^99m^Tc-HP-Ark2 SPECT/CT

**Fig. 5:**
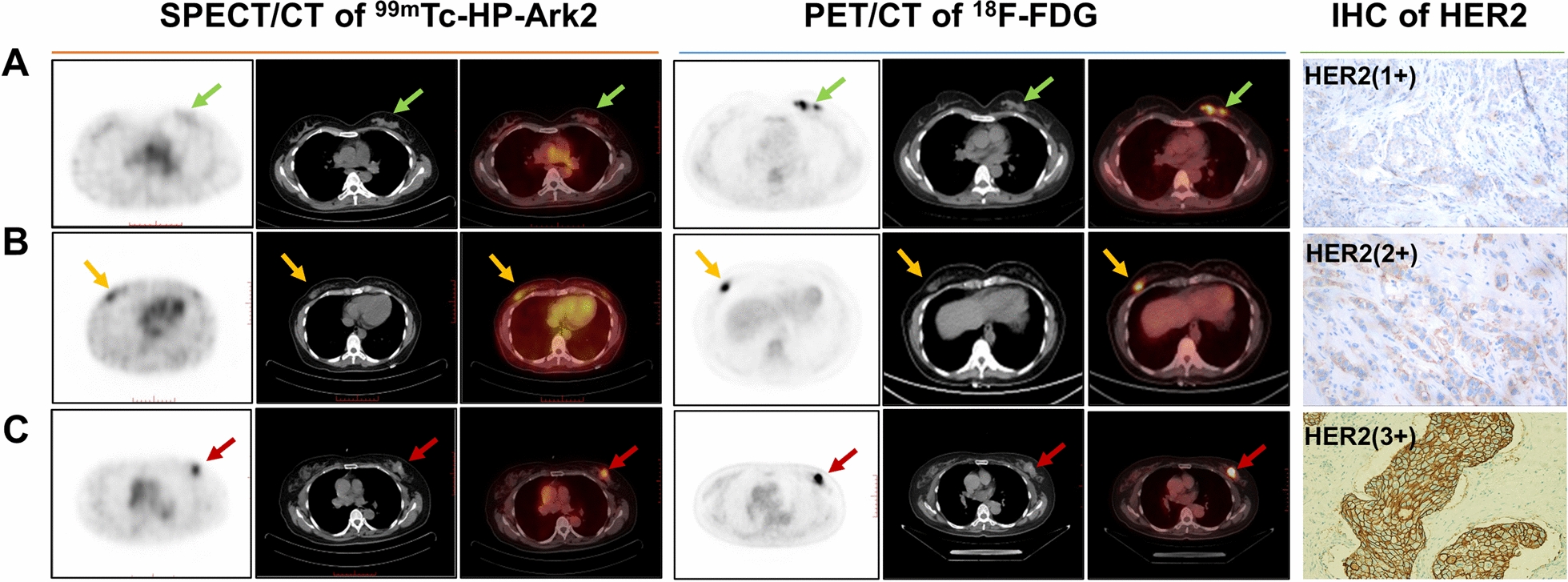
Representative ^99m^Tc-HP-Ark2 SPECT/CT images (transaxial view of SPECT, CT and SPECT/CT fusion), ^18^F-FDG PET/CT images (transaxial view of PET, CT and PET/CT fusion), and HER2 immunohistochemical (IHC) stains of three patients with breast cancer. **A** A 33-y-old woman (Patient No. 6) with HER2 (1 +) invasive ductal cancer in her left breast (green arrows), ^99m^Tc-HP-Ark2 tumor-to-background ratio, T/B = 1.00 and ^18^F-FDG T/B = 11.60. **B** A 39-y-old woman (Patient No. 1) with HER2 (2 +) invasive lobular cancer in her right breast (brown arrows), ^99m^Tc-HP-Ark2 T/B = 1.64 and ^18^F-FDG T/B = 15.64. **C** A 57-y-old woman (Patient No. 26) with HER2 (3 +) invasive ductal cancer in her left breast (red arrows), ^99m^Tc-HP-Ark2 T/B = 4.22 and.^18^F-FDG T/B = 6.77

After the safety evaluation (see Additional file 1: Figure S8-S9 and Table S1 in supplementary material) and ethics approval, the pilot clinical application of ^99m^Tc-HP-Ark2 SPECT/CT was performed in 34 patients (Table 1). No adverse events related to tracer administration were observed. The quantified biodistribution of ^99m^Tc-HP-Ark2 in all patients was acquired (Additional file 1: Figure S10). The results demonstrated that the kidneys, as the main metabolic pathway, showed a high uptake, and the uptake of other organs was relatively low, which was consistent with the results of preclinical studies. Representative images as well as the HER2 status of HER2 (1 +), HER2 (2 +) and HER2 (3 +) breast cancer patients were shown in Fig. 5. For patient No. 6 (Fig. 5A), SPECT/CT images showed little uptake of ^99m^Tc-HP-Ark2 in the left breast lesion (T/B = 1.00); in contrast, the PET/CT images showed intense uptake of ^18^F-FDG in this lesion (T/B = 11.60). SPECT/CT imaging was more consistent with the IHC score HER2 (1 +). For patient No. 1 (Fig. 5B), SPECT/CT images showed moderate uptake of ^99m^Tc-HP-Ark2 in the right breast lesion (T/B = 1.64), while the PET/CT images showed intense uptake of ^18^F-FDG in the lesion (T/B = 15.64), and IHC scored HER2 (2 +). In Fig. 5C, SPECT/CT images displayed intense tracer accumulation in the left breast tumor of patient No. 26 with IHC classified HER2 (3 +), with T/B of 4.22, while PET/CT images showed moderate uptake of ^18^F-FDG in the left breast lesion (T/B = 6.77).

**Table 1:** Basic characteristics of the enrolled patients (n = 34)

Patient No	Age	Pathologic diagnosis	IHC	FISH	^99m^Tc-HP-Ark2 SPECT/CT					^18^F-FDG PET/CT				
					Tumor		Breast tissue		T/B ratio	Tumor		Breast tissue		T/B ratio
					Counts_avg_	Counts_max_	Counts_avg_	Counts_max_		SUV_mean_	SUV_max_	SUV_mean_	SUV_max_	
1	39	ILC	2 +	−	141	154	86	95	1.64	6.1	7.6	0.39	0.56	15.64
2	63	IDC	1 +		91	104	85	104	1.07	0.8	1.1	0.5	0.6	1.6
3	56	IDC	0		55	64	40	54	1.38	2.7	3.5	0.6	0.8	4.5
4	52	MeC	3 +		143	171	77	87	1.86	5.3	8.1	0.3	0.8	17.67
5	60	IDC	2 +	+	186	209	102	123	1.82	5.4	8.4	0.7	0.9	7.71
6	33	IDC	1 +		65	85	65	87	1	5.8	7.8	0.5	0.8	11.6
7	41	IDC	2 +	+	227	245	93	107	2.44	8.2	11.6	1.1	1.6	7.45
8	38	MuC	1 +		71	91	87	115	0.82	1.6	2.3	0.9	1.2	1.78
9	42	IDC	1 +		80	106	60	78	1.33	7.9	9.1	1.2	1.7	6.58
10	32	IDC	3 +		131	157	82	122	1.6	13.5	19.6	1.5	1.9	9
11	45	IDC	1 +		112	133	88	99	1.27	3.2	4.5	0.9	1.2	3.56
		IDC	1 +		121	128	88	99	1.38	2.5	4.3	0.9	1.2	2.78
12	38	IC	1 +		187	210	177	221	1.06	2.7	3.7	1.8	1.2	1.5
13	53	IDC	1 +		170	200	128	142	1.33	3.3	4.1	0.3	0.4	11
14	58	IDC	2 +		132	154	55	61	2.4	7.6	9.1	0.5	0.7	15.2
15	50	IDC	2 +	+	40	50	24	32	1.67	3.7	5.5	1.8	2.3	2.06
16	54	IC	2 +	−	109	116	82	87	1.33	2.6	3.4	0.6	0.8	4.33
17	36	ILC	1 +		87	115	123	170	0.71	1.5	1.8	1.5	1.9	1
18	49	IDC	2 +	−	217	219	191	211	1.14	3.2	4.3	1.6	2.3	2
19	48	IDC	3 +		170	202	105	123	1.62	5.3	7.8	1.5	2	3.53
20	60	IDC	3 +		243	294	83	93	2.93	6.2	8.5	0.5	0.9	12.4
21	45	IDC	2 +	+	264	293	71	103	3.72	6	9.5	0.3	0.6	20
22	61	IDC	2 +	−	289	310	60	65	4.82	7.7	10.2	0.8	0.9	9.63
23	38	IDC	1 +		223	256	137	141	1.63	7.8	10.5	1.5	1.8	5.2
24	53	IDC	3 +		259	292	84	100	3.08	3.5	5	0.6	0.8	5.83
25	38	IDC	1 +		135	153	104	116	1.3	1.5	2.2	1.1	1.6	1.36
		IDC	1 +		128	141	104	116	1.23	1.6	2.1	1.1	1.6	1.45
26	57	IDC	3 +		359	400	85	95	4.22	8.8	12.9	1.3	1.5	6.77
27	54	IDC	3 +		220	237	107	122	2.06	5.2	6.8	1.1	1.3	4.73
28	42	IDC	1 +		161	172	193	206	0.83	1.6	1.8	1.1	1.2	1.45
29	58	IDC	3 +		197	206	65	72	3.03	6.6	10	1.1	1.2	6
30	48	MuC	0		130	137	168	210	0.77	1.1	1.9	1.1	1.4	1
31	67	IDC	0		133	165	80	96	1.66	2.7	3.7	0.5	0.5	5.4
32	34	IDC	1 +		120	135	125	135	0.96	2.6	3	0.8	1	3.25
33	60	IDC	2 +	−	387	435	86	124	4.5	7.3	8.3	1.5	1.9	4.87
34	55	IDC	2 +	−	141	156	94	106	1.5	6.6	9.6	1.3	1.6	5.08

*IHC* immunohistochemistry, *FISH* fluorescence in situ hybridization, *T/B* ratio of tumor-to-background (breast), *Counts*_*avg*_ average counts, *Counts*_*max*_ maximum counts, *SUV*_*mean*_ mean standardized uptake value, *SUV*_*max*_ maximum standardized uptake value, *ILC* invasive lobular carcinoma, *IDC* invasive ductal carcinoma, *MeC* medullary carcinoma; IC: intraductal carcinoma; MuC: mucinous carcinoma

**Fig. 6:**
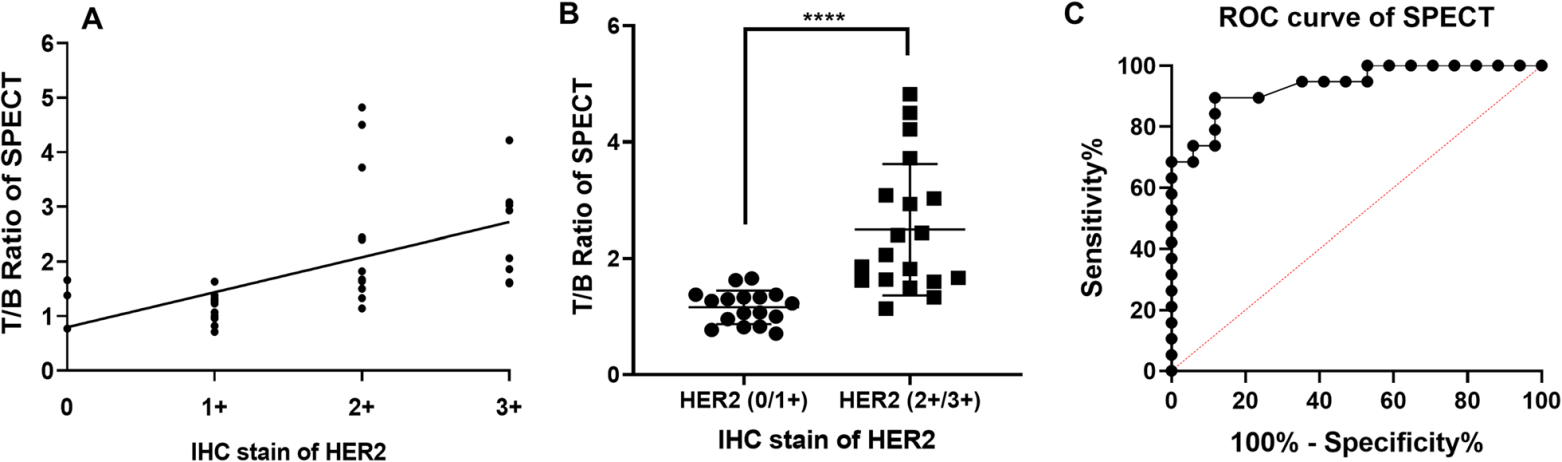
Comparison of T/B ratios and IHC scores. **A** Correlation between the SPECT T/B ratio and IHC score. **B** Comparison between the T/B ratio of HER2 (2 + /3 +) and HER2 (0/1 +) tumors. **C** ROC analysis

Among the 36 primary lesions in the 34 patients, 17 HER2 (0/1 +) lesions in 15 patients were classified as negative, and 11 HER2 (2 +) and 8 HER2 (3 +) lesions in 19 patients were classified as positive for HER2 expression. The correlation between the ^99m^Tc-HP-Ark2 SPECT T/B ratio and the HER2 expression level of breast cancer was shown in Fig. 6. There was a moderate linear correlation between the SPECT T/B ratio and the IHC score, according to Spearman’s rank correlation test (r = 0.691, P < 0.0001). ^99m^Tc-HP-Ark2 SPECT imaging significantly differentiated HER2-positive tumors (T/B ratio 2.49 ± 1.13, n = 19) from negative tumors (1.16 ± 0.29, n = 17) (P < 0.0001). A receiver operating characteristic curve (ROC) analysis was performed to determine the optimal cutoff value to discriminate HER2 (2 + /3 +) versus HER2 (0/1 +) tumors. The ROC analysis showed that the area under the curve (AUC) was 0.935. The optimal cutoff value was 1.44, at which the sensitivity, specificity, and accuracy were 89.5% (17/19), 88.2% (15/17), and 88.9% (32/36), respectively. For ^18^F-FDG PET imaging, the T/B ratio of HER2 (2 + /3 +) tumors (8.42 ± 5.33, n = 19) was also significantly higher than that of HER2 (0/1 +) tumors (3.82 ± 3.29, n = 17) (P = 0.0051). The ROC analysis showed that the area under the curve (AUC) was 0.802. The optimal T/B cutoff value for PET was 4.62, at which the sensitivity, specificity, and accuracy were 78.9% (15/19), 70.6% (12/17), and 75.0% (27/36), respectively (Additional file 1: Figure S11). In addition, the positive predictive value (PPV) and negative predictive value (NPV) of ^99m^Tc-HP-Ark2 SPECT were 89.5% (17/19) and 88.2% (15/17), respectively, compared with 75.0% (15/20) and 75.0% (12/16) for ^18^F-FDG PET.

For the 11 cases with a HER2 (2 +) tumor, FISH was further performed. Four FISH + tumors were classified as HER2-positive, whereas the 7 FISH- tumors were classified as HER2-negative. Using IHC (2 +) plus FISH + or IHC (3 +) as a positive evaluation criterion, ^99m^Tc-HP-Ark2 SPECT imaging significantly differentiated HER2-positive tumors (T/B ratio 2.50 ± 0.88, n = 12) from negative tumors (1.54 ± 1.03, n = 24) (P = 0.0092). The ROC analysis of ^99m^Tc-HP-Ark2 SPECT yielded an AUC of 0.875. The optimal cutoff value (T/B ratio) for ^99m^Tc-HP-Ark2 SPECT was 1.55, at which the sensitivity, specificity, and accuracy were 100.0% (12/12), 75.0% (18/24), and 83.3% (30/36), respectively (Additional file 1: Figure S12 A-B). ^18^F-FDG PET imaging was not effective in distinguishing HER2-positive tumors (T/B ratio 8.60 ± 5.48, n = 12) from HER2-negative tumors (5.07 ± 4.40, n = 12) under this criterion (P = 0.068), and the sensitivity, specificity, and accuracy were 75.0% (9/12), 75.0% (18/24), and 75.0% (27/36), respectively (Additional file 1: Figure S12 C-D). In addition, the PPV and NPV of ^99m^Tc-HP-Ark2 SPECT were 66.7% (12/18) and 100% (18/18), respectively, compared with 60.0% (9/15) and 85.7% (18/21) for ^18^F-FDG PET.

### Detection of lymph node metastases

**Fig. 7:**
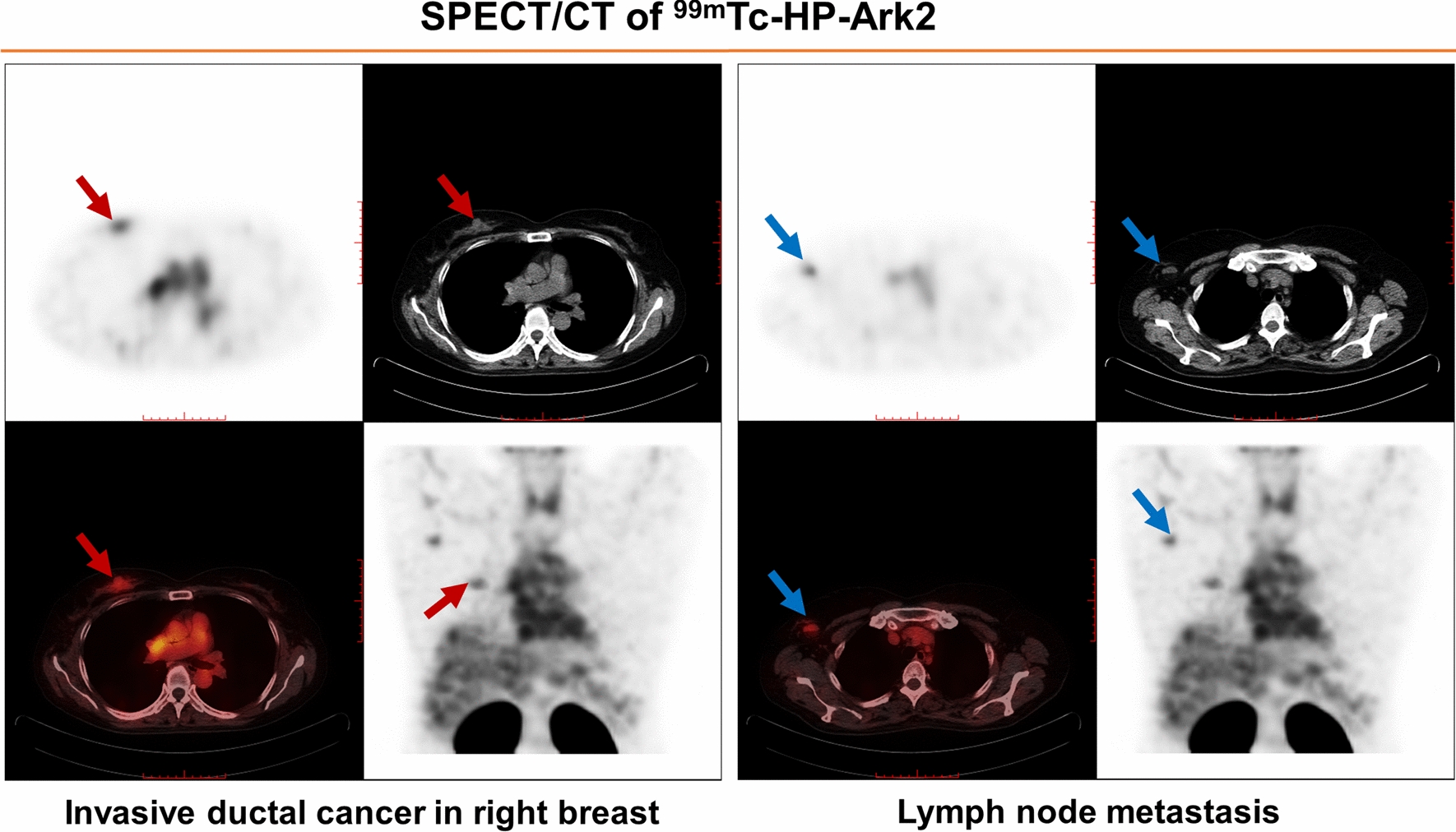
Representative SPECT/CT images of a 58-y-old woman (Patient No. 29) with invasive ductal cancer in her right breast and right axillary lymph node metastasis. The transverse plane SPECT, CT, SPECT/CT fusion and SPECT maximal intensity projection (MIP) images showed intense ^99m^Tc-HP-Ark2 uptake in both the breast tumor (red arrow) and axillary lymph node metastasis (blue arrows)

All 34 patients underwent surgery and pathological examination, which confirmed that 20 patients had lymph node metastases. IHC was not performed on lymph nodes. Among these 20 patients, ^99m^Tc-HP-Ark2 SPECT/CT detected metastatic lymph nodes in all 6 patients (Nos. 4, 5, 7, 15, 24 and 29) with HER2-positive tumors (3 patients with IHC (3 +) tumors and 3 patients with IHC (2 +) plus FISH + tumors) who had ipsilateral lymph node metastases. Representative SPECT/CT images of patient No. 29, a 58-y-old woman with invasive ductal cancer in the right breast whose IHC score was HER2 (3 +), are shown in Fig. 7. Both a breast tumor and ipsilateral axillary lymph node metastasis were clearly visualized. ^18^F-FDG PET could detect metastases in all patients regardless of whether they were HER2-positive.

## Discussion

Assessment of HER2 expression is critical for identifying breast cancer patients who may benefit from HER2-targeted therapy and monitoring their response to treatment [28]. Due to the temporal and spatial heterogeneity of HER2 expression in breast tumors, IHC and FISH might not accurately reflect the HER2 status in real time, which may lead to a misguided treatment decision [29, 30]. Molecular imaging provides a noninvasive, real-time, dynamic tool for the clinical detection of HER2 expression. ^18^F-FDG PET/CT is the most commonly used tumor detection method in nuclear medicine. Studies have shown that the uptake of ^18^F-FDG in primary breast tumors is correlated with its histopathological and molecular characteristics, and it also shows differential uptake in some HER2-positive and HER2-negative tumors [31, 32]. It was also used to predict the outcome of neoadjuvant therapy and identified poor metabolic responders who are at high risk of residual tumor after two cycles of chemotherapy plus trastuzumab [33]. However, ^18^F-FDG PET/CT imaging cannot directly reflect the status of HER2, so it cannot be effectively used for guidance of HER2-targeted therapy. Although preliminary clinical studies have been carried out on some HER2-targeted nanobody and affibody probes, their preparation is relatively troublesome and requires purification, which has certain technical requirements for clinical operators and is not conducive to widespread clinical promotion. The preparation of peptide probes is relatively simple, but clinical research on HER2-targeted peptide molecular probes is still scarce [15]. We previously developed the HER2-targeted molecular probe ^99m^Tc-H10F, which bound to different binding site of HER2 (extracellular II vs. extracellular IV) with trastuzumab, but preliminary clinical trial revealed that its uptake in tumors was insufficient and limited its further clinical translation [27]. Based on the H10F peptide, a novel HER2-specific imaging probe, ^99m^Tc-HP-Ark2, was developed for clinical practice, and a comparative study on ^99m^Tc-HP-Ark2 SPECT/CT and ^18^F-FDG PET/CT was performed for the detection of HER2 expression in breast cancer patients. The results demonstrated that HER2-targeted SPECT/CT imaging with ^99m^Tc-HP-Ark2 could noninvasively reflect the status of HER2 in breast cancer.

The tumor-targeting ability of a probe comes largely from the binding affinity of its targeting molecule to the target. As shown in our SPR results, the retro-inverse D-peptide rk possessed the same level of binding affinity toward HER2 protein as the parental L-peptide H10F, mainly due to the similar stereochemical structure between rk and H10F [34–36]. The rk peptide could also distinguish HER2 from other EGFR family proteins, including EGFR, HER3 and HER4, demonstrating the specific targeting capability of the rk peptide to HER2 protein. Tri-amino acid sequences, hydrocarbons, and PEG linkers have been used as specific pharmacokinetic modifiers to improve the pharmacokinetic characteristics of probes [37, 38]. Among these linkers, the hydrocarbon Aoc linker can enhance probe retention in tumors. Here, two Aoc linkers were introduced as flexible linking chains between dimerized rk entities to raise the chances that the probe would bivalently bind to HER2, contributing to increased binding affinity. The lipophilic Aoc also increased the retention of the probe in the tumors, contributing to improved imaging quality. Dimerization has been widely used to improve the binding affinity of monomer peptides [39–41]. The binding affinity of the Ark2 dimer peptide was significantly higher (almost 8 times) than that of the rk monomer peptide. In addition, since the d amino acids in the peptide backbone cannot be recognized by common proteases in the body, the metabolic stability of ^99m^Tc-HP-Ark2 in vivo was significantly improved. These optimizations significantly enhanced the tumor uptake of ^99m^Tc-HP-Ark2 and improved the imaging quality of SPECT/CT, which enabled ^99m^Tc-HP-Ark2 SPECT/CT to detect small tumors, enhancing its clinical application value. Moreover, the tumor uptake of ^99m^Tc-HP-Ark2 showed a significant linear correlation with the HER2 expression level, showing its good prospects for HER2 detection in clinical practice.

In this pilot prospective clinical study, when IHC (2 +) or IHC (3 +) was used as the criterion for positivity, ^99m^Tc-HP-Ark2 SPECT/CT imaging with a T/B ratio of 1.44 as the cutoff value specifically reflected the HER2 status, with a sensitivity of 89.5%, specificity of 88.2% and accuracy of 88.9%. Overall, ^99m^Tc-HP-Ark2 SPECT/CT and IHC were fairly effective in detecting tumor HER2 expression, despite differences in overall tumor evaluation and local tumor biopsy analysis. Although PET imaging also showed a significant difference in ^18^F-FDG uptake between HER2-positive and HER2-negative tumors, it was based on the reflection of the metabolic level, so that the sensitivity (75.0%), specificity (78.9%) and accuracy (70.6%) of its detection were obviously inferior to those of specific ^99m^Tc-HP-Ark2 SPECT imaging. In particular, 100% of IHC (3 +) tumors were identified by ^99m^Tc-HP-Ark2 SPECT/CT imaging. Therefore, ^99m^Tc-HP-Ark2 SPECT/CT can be expected to replace IHC in some situations where biopsy is inconvenient or impossible. When IHC (2 +) plus FISH + or IHC (3 +) was used as the criterion for HER2 positivity, ^99m^Tc-HP-Ark2 SPECT/CT imaging with a T/B ratio of 1.55 as the cutoff value still reflected the corresponding HER2 test results with a sensitivity of 100%, specificity of 75% and accuracy of 83.3%. The tumors with IHC (3 +) or IHC (2 +) plus FISH + were all accurately detected by ^99m^Tc-HP-Ark2 SPECT/CT imaging, while 4 tumors that were IHC (2 +) plus FISH- showed inconsistent results. Under this criterion, there was no significant difference in ^18^F-FDG uptake between HER2-positive and HER2-negative tumors, and its detection sensitivity, specificity, and accuracy were also inferior to those of specific SPECT imaging, all of which were 75.0%. The ^99m^Tc-HP-Ark2 SPECT/CT imaging results did not correlate with FISH results in some IHC (2 +) tumors, possibly because ^99m^Tc-HP-Ark2 targets HER2 at the protein level rather than the gene level. Due to the heterogeneity of HER2 expression, individual pathological sections often cannot reflect the overall expression of HER2 in breast tumors.

Many studies have revealed HER2 expression heterogeneity not only between primary and metastatic lesions but also between metastatic lesions [22, 42]. Since the efficacy of trastuzumab is highly dependent on the HER2 status of tumors, if the metastases of HER2-positive tumors are not HER2-positive, it would make targeted therapy harder [43]. The European guidelines have already recommended the biopsy of metastases to reassess the HER2 status [44], but in clinical practice, this is not easy, and it is impossible when multiple metastases exist. HER2-specific molecular imaging can not only comprehensively evaluate the expression of HER2 in tumors in situ but also reflect HER2 expression in metastatic lesions; therefore, ^99m^Tc-HP-Ark2 SPECT/CT is of great significance for guiding targeted therapy. Among 20 patients with metastatic lymph nodes, only 6 patients with HER2-positive tumors, including 3 patients with IHC (3 +) tumors and 3 patients with IHC (2 +) plus FISH + tumors, had ipsilateral lymph node metastases. The primary tumors and metastatic lymph nodes were successfully detected by ^99m^Tc-HP-Ark2 SPECT/CT imaging. Among the other 14 patients, 4 patients’ primary lesions were HER2-positive, and 10 patients’ primary lesions were HER2-negative. Since the IHC test was not performed for the lymph nodes, the HER2 status of metastatic lymph nodes was not clear. This was a limitation of this study. In addition, the current resolution of clinical SPECT/CT might limit the detection of small metastatic lesions.

HER2 downregulation is an indicator of the effectiveness of trastuzumab [8, 9]. Since ^99m^Tc-HP-Ark2 has no cross-interference with trastuzumab, it can be used to evaluate the therapeutic efficacy of trastuzumab during treatment. Our previous preclinical study revealed that ^99m^Tc-H10F SPECT/CT imaging could reflect the effectiveness of trastuzumab treatment earlier than the tumor volume change in animal models [27]. Since the purpose of this pilot clinical study was to verify the feasibility of ^99m^Tc-HP-Ark2 SPECT/CT for the accurate and noninvasive detection of HER2 expression in breast cancer, the investigation of monitoring therapy was not performed in breast cancer patients. In addition, we also wondered whether the tumor uptake level of ^99m^Tc-HP-Ark2 could indicate the effectiveness of trastuzumab treatment before therapy, as well as whether the HER2 expression change during the therapy would be related to drug resistance to adjust the ongoing trastuzumab treatment. We hope to answer these questions in future clinical studies.

## Conclusion

We have developed a novel HER2-targeted SPECT imaging probe, ^99m^Tc-HP-Ark2. The kit formulation is simple, efficient, and reproducible, making it very convenient for routine clinical use. ^99m^Tc-HP-Ark2 showed enhanced tumor uptake, improved pharmacokinetic properties and increased imaging contrast over the previous ^99m^Tc-H10F probe, which made ^99m^Tc-HP-Ark2 more suitable for clinical application. The pilot study in 34 breast cancer patients showed that ^99m^Tc-HP-Ark2 SPECT/CT could noninvasively reflect the status of HER2 in breast cancer, which showed great potential to identify patients to receive trastuzumab treatment and monitor the therapeutic efficacy earlier during trastuzumab treatment. This prospective clinical study merits ^99m^Tc-HP-Ark2 SPECT/CT for further clinical validation in larger cohorts.

## Materials and methods

### Materials

The peptides KLRLEWNR (H10F), _D_(RNWELRLK) (rk), 8-Aminooctanoic-_D_(RNWELRLK) (Ark), Glu[8-aminooctanoic-_D_(RNWELRLK)]_2_ (Ark2) and the corresponding sulfhydryl peptides (Cys-H10F, Cys-rk and Cys-Ark2) and Cy5-Ark2 peptide were purchased from GL Biochem Ltd. ^99m^Tc-pertechnetate was eluted from a commercial ^99^Mo/^99m^Tc generator (Beijing Atomic High-Tech Co., Ltd., Beijing, China). All compounds had > 95% purity and were directly used in experiments. The pharmacokinetic modifying linker PEG_4_ was purchased from Xian Ruixi Biological Technology Co., Ltd. Sodium succinimidyl 6-(2-(2-sulfonatobenzaldehyde) hydrazono) nicotinate (HYNIC-OSu) was prepared as described [45].

### Cell culture and animal models

The SK-BR-3, MCF7 and MDA-MB-468 human breast cancer cell lines and HT29 human colorectal cancer and BxPC3 human pancreatic cancer cell lines were obtained from the Cell Resource Center, Peking Union Medical College (which is the headquarters of the National Infrastructure of Cell Line Resource, NSTI, Beijing, China). The cell lines were confirmed to be free of mycoplasma contamination by polymerase chain reaction (PCR) and culturing and were authenticated by short tandem repeat (STR) profiling (FBI, CODIS, http://cellresource.cn).

The MCF7-HER2 cell line with stable expression of HER2 was established by our laboratory. The HER2 gene was amplified by PCR and cloned into the lentiviral vector pCDH. The recombinant lentiviral vectors (psPAX2, pMD2G) and pCDH-HER2 were cotransfected into 293 T cells, and the recombinant lentiviral supernatant was used to infect MCF7 cells. Promycin was used to screen the cell lines stably expressing HER2. MCF7-HER2 cells were identified by flow cytometry and western blotting. SK-BR-3 and HT29 cells were grown in McCoy's 5A medium, MCF7 and MCF7-HER2 cells were grown in high-glucose Dulbecco’s modified Eagle medium (Macgene, Beijing), MDA-MB-468 cells were grown in Leibovitz's L-15 medium, and BxPC3 cells were grown in RPMI-1640 medium. All cell lines were cultured in medium supplemented with 10% fetal bovine serum (FBS) at 37 °C in a humidified atmosphere containing 5% CO_2_.

To establish subcutaneous tumor models, SK-BR-3 and MDA-MB-468 cells (1 × 10^7^), MCF7 and MCF7-HER2 cells (5 × 10^6^), or HT29 and BxPC3 cells (3 × 10^6^) in 200 μL of phosphate-buffered saline (PBS) were inoculated subcutaneously into female NOD SCID mice. The animals were randomly chosen for in vivo assays when the tumor size reached 100–150 mm^3^ (1–2 weeks after inoculation). For the establishment of an orthotopic breast cancer model, 3 × 10^6^ SK-BR-3 cells were injected into the female NOD SCID mice’s chest wall breast fat pad, and the mice were submitted to in vivo imaging when the tumor volume reached 20–50 mm^3^ (approximately 2 weeks). All animal experiments were performed by following the protocol approved by the Institutional Animal Care and Use Committee at Peking University.

### Flow cytometry

To assess HER2 expression in SK-BR-3, MCF7, MCF7-HER2, HT29, BxPC3 and MDA-MB-468 cells, samples were harvested and suspended in PBS, followed by incubation with 20 nM PE-conjugated anti-human CD340 (erbB2/HER2) antibody (Biolegend, USA) at 4 °C for 1 h. Then, the cells were washed with cold PBS and analyzed in a BD LSRII flow cytometer (Becton Dickinson, USA). The results were analyzed using FlowJo, version 10.

### Surface plasmon resonance (SPR)

To compare the affinities with which different peptides bound to HER2, SPR analyses were performed on a Plexera PlexArray HT system (Plexera LLC, Bothell, WA) using a bare gold SPR chip (Nanocapture gold chips, with a gold layer of 47.5 nm thickness). All sulfhydryl peptides (Cys-H10F, Cys-rk and Cys-Ark2) were printed onto the gold chip surface by using the thiol group of the cysteine residue. The printed chip was then incubated at 4 °C overnight in a humid box. The SPR chip was washed and blocked using 5% nonfat milk in PBS overnight before use. The SPR analytical procedure involved the following cycle of injections: running buffer (PBS, baseline stabilization); sample (six concentrations of the protein, binding); running buffer (PBS, washing); and 0.5% (v/v) H_3_PO_4_ in deionized water (regeneration). HER2 protein was diluted with PBS to concentrations of 70.4 nM, 35.2 nM, 17.6 nM, and 8.8 nM. Real-time binding signals were recorded and analyzed by PlexArray HT software. Other proteins in the EGFR family, including EGFR, HER3, and HER4, were utilized as controls with the same procedure above.

### In vitro cell staining

To evaluate the HER2-binding capability of the Ark2 peptide, fluorescence staining of Cy5-Ark2 in HER2-positive SK-BR-3 and HER2-negative MCF7 cells was performed. Approximately 1 × 10^5^ cells per mL were seeded into culture dishes and cultured overnight for cell adherence. Cy5-Ark2 was dissolved in cold PBS at a concentration of 50 μM. To verify the specific binding of Ark2 peptide to SK-BR-3 cells, a self-blocking experiment was carried out by incubating the cells with 200 µL (50 μM) Cy5-Ark2 and a 200-fold excess of cold rk peptide at 4 °C for 1 h. To assess the interaction between Ark2 and trastuzumab (Herceptin^®^, Genentech, Inc., USA), the cross-blocking assay was carried out by incubating SK-BR-3 cells with 200 µL (50 μM) Cy5-Ark2 and 200-fold excess of trastuzumab at 4 °C for 1 h.

### Synthesis of HP-Ark2 conjugate

Ark (Dde) and Boc-Glu(OSu)-OSu were dissolved in 500 μL N,N-dimethylformamide (DMF) and mixed with 3 μL N,N-diisopropylethylamine (DIEA). After stirring for 8 h at room temperature, conjugate (Boc)Glu-Ark(Dde)2 was isolated by semipreparative high-performance liquid chromatography (HPLC). Fractions containing the product were collected and lyophilized. (Boc)Glu-Ark(Dde)2 was dissolved in 1 mL trifluoroacetic acid (TFA) and stirred for 10 min at room temperature. Conjugate Glu-Ark (Dde)2 was blown dry by nitrogen.

NH_2_-PEG_4_-COOH and HYNIC-OSu were dissolved in 500 μL DMF and mixed with 3 μL DIEA. After stirring for 8 h at room temperature, the HYNIC-PEG_4_-COOH conjugate was isolated by semipreparative HPLC. Fractions containing the product were collected and lyophilized. HYNIC-PEG_4_-COOH was dissolved in 500 μL DMF and mixed with 1-(3-dimethylaminopropyl)-3-ethylcarbodiimide hydrochloride (EDC) and N-hydroxysuccinimide (NHS). After stirring for 8 h at room temperature, the HYNIC-PEG_4_-OSu conjugate was isolated by semipreparative HPLC. Fractions containing the product were collected and lyophilized.

HYNIC-PEG_4_-NHS and Glu-Ark(Dde)2 were dissolved in 500 μL DMF and mixed with 3 μL DIEA. After stirring for 8 h at room temperature, the HYNIC-PEG_4_-Ark(Dde)2 conjugate was isolated by semipreparative HPLC. Fractions containing the product were collected and lyophilized. HYNIC-PEG_4_-Ark(Dde)2 was dissolved in 500 μL 2% hydrazine hydrate and stirred for 30 min at room temperature. The conjugate HYNIC-PEG_4_-Ark2 (termed HP-Ark2) was isolated by semipreparative HPLC and lyophilized.

### Preparation of ^99m^Tc-labeled tracers

^99m^Tc-HP-Ark2 was prepared via a kit formulation. Briefly, 50 µg HP-Ark2, 6.5 mg N-[tris(hydroxymethyl)methyl]glycine (tricine) and 5.0 mg trisodium triphenylphosphine-3,3′,3′′-trisulfonate (TPPTS) were dissolved in 1.0 mL pH 4.7 (0.1 M) succinate buffer and lyophilized as a labeling kit. Approximately 1.0 mL of Na^99m^TcO_4_ solution (555–925 MBq) in saline was added to the labeling vial. The vial was heated in a lead-shielded boiling water bath for 20 min. ^99m^Tc-HP-rk and ^99m^Tc-H10F were prepared with the same radiolabeling procedure. A sample of the resulting solution was subjected to radioactive high-performance liquid chromatography (radio-HPLC) for radiochemical purity (RCP) determination. The RCP of the tracer was required to be > 95% for further use.

### Small-animal SPECT/CT imaging

First, small-animal SPECT/CT imaging of ^99m^Tc-HP-Ark2 was performed in female NOD SCID mice bearing SK-BR-3 human breast cancer xenografts. Each tumor-bearing mouse was injected via the tail vein with ~ 37 MBq (~ 2.5 µg) of ^99m^Tc-HP-Ark2. At 0.5, 1 and 2 h p.i., the mice were anesthetized by inhalation of 2% isoflurane and imaged using a nanoScan SPECT/CT system (Mediso Inc., Hungary) following a standard protocol [46]. Briefly, the small-animal SPECT images were acquired with the technetium-99 m parameters (peak 140 keV with 20% width, frame time 25 s), and CT images were acquired with default settings (50 kVp, 0.67 mA, and rotation 210°, exposure time 300 ms). All SPECT/CT images were reconstructed and further analyzed by Fusion software (Mediso Inc., Hungary), including drawing volumes of interest on tumors and major organs as well as calculating the tumor uptake (%ID/cc) accordingly. Blocking was studied by coinjection of ^99m^Tc-HP-Ark2 with a blocking dose of (25 mg/kg) cold rk peptide or (30 mg/kg) trastuzumab, and nanoScan SPECT/CT imaging was performed at 0.5 h p.i. Small-animal imaging of the monomer tracer ^99m^Tc-HP-rk was also conducted as a control. The in vivo behavior of ^99m^Tc-HP-Ark2 in MCF7, MCF7-HER2, HT29, BxPC3 and MDA-MB-468 tumor xenograft models was also evaluated by nanoScan SPECT/CT imaging at 0.5 h p.i., following the above standard protocol.

### Biodistribution

The biodistribution experiment of ^99m^Tc-HP-Ark2 was performed in the SK-BR-3 tumor xenograft model. Each mouse was injected with 0.37 MBq (~ 0.03 µg) of tracer to evaluate the distribution of the probe in tumors and major organs (4 mice/group). The mice were sacrificed and dissected at 0.5, 1 and 2 h p.i. Blood, tumor, liver, kidney and other major organs were collected and weighed, and their radioactivity was counted in a Wallac 1480 γ-counter (Perkin-Elmer, Waltham, MA). For the blocking group, excess (300 µg) cold rk peptide was coinjected with 0.37 MBq (0.03 µg) of ^99m^Tc-HP-Ark2. The biodistributions of ^99m^Tc-HP-Ark2 and ^99m^Tc-HP-rk in the SK-BR-3 tumor model were also compared, and MCF7 and MDA-MB-468 tumor xenograft models were used as controls. All biodistribution data are presented as the percentage of injected dose per gram of tissue (%ID/g).

### In vivo stability

NOD SCID normal mice were used for metabolism assays as previously described [47]. Briefly, each mouse was administered ~ 37 MBq (~ 2.5 µg) of ^99m^Tc-HP-Ark2 via the tail vein. Urine samples were collected at 2 h p.i. and mixed with an equal volume of 50% acetonitrile aqueous solution. The mixture was centrifuged at 8,000 rpm for 5 min at room temperature. The supernatant was collected and passed through a 0.22 μm Millex-LG filter unit to remove any precipitate or particles. The filtrate was analyzed by radio-HPLC. The metabolic stability of ^99m^Tc-H10F was evaluated by the same procedure for comparison.

### Dosimetry estimation

The dosimetry calculation was performed according to the European Association of Nuclear Medicine Dosimetry Guidance and calculated using OLINDA/EXM (version 1.1; Vanderbilt University). The radiation effective dose (mSv/MBq) of the main organs of the human body was estimated from the biodistribution data of the SK-BR-3 tumor models (Additional file 1: Table S1). The calculated mean effective dose equivalent for the whole body was 9.17 × 10^–3^ mSv/MBq for males and 1.03 × 10^–2^ mSv/MBq for females.

### Clinical SPECT/CT and PET/CT imaging

The clinical trial was designed and approved by the ethnic committee of Peking Union Medical College Hospital (protocol JS2074) and registered online at *ClinicalTrials.gov* (NCT04267900). Written informed consent was obtained from each patient before the study. A total of 34 female patients (mean age ± SD: 49 ± 10 y) suspected of having breast cancer according to mammography or ultrasonography were recruited from December 7th, 2019, through December 31th, 2020, at Peking Union Medical College Hospital (Inclusion Criteria: patients in suspicion of breast cancer by mammography or ultrasonography, and being able to provide basic information and sign the written informed consent form. Exclusion Criteria: the exclusion criteria included claustrophobia, pregnancy, breastfeeding, kidney or liver failure, inability to fulfill the study, and undergoing any preceding local or systemic therapies that might interfere with HER2 binding.). The patients’ basic information is listed in Table 1. Within 1 week before surgery, the patients first underwent ^18^F-FDG PET/CT imaging on a time-of-flight PET/CT scanner (Polestar m660, SinoUnion Healthcare Inc., China) 1 h after intravenous injection of ^18^F-FDG at a dose of approximately 5.6 MBq (0.15 mCi) per kilogram body weight. SPECT/CT imaging was acquired with a Philips Precedence scanner (Philips Healthcare, Andover, Massachusetts, USA) within 3 days after PET imaging 0.5 h after intravenous injection of ^99m^Tc-HP-Ark2 at a dose of approximately 11.1 MBq (0.3 mCi) per kilogram body weight, ranging from 399.6 to 832.5 MBq (mean, 639.1 MBq) per patient. The wrist or elbow on the same side as the healthy breast was selected for injection. All images were measured and quantified blindly and separately by two experienced nuclear medicine physicians (Zhaohui Zhu and Rongxi Wang). On PET images, the volumes of interest (VOIs) were drawn over tumors using a 40% threshold, and the software automatically obtained the radioactivity concentration and the maximum and the mean standardized uptake values (SUV_max_ and SUV_mean_, respectively). T/B ratios were calculated from SUV_mean_ values for further analysis. The contralateral breast tissue was considered the background for calculations. For semiquantitative analyses of SPECT images, the same VOI method as above was adopted to obtain the maximum and the average count values (Counts_max_ and Counts_avg_, respectively) of the breast tumors. The Counts_avg_ value was compared between the tumor region and the contralateral region to obtain the T/B ratio. Calculations were performed using SPSS 23.0 software (IBM SPSS, Chicago, IL, USA) and GraphPad Prism 8 (GraphPad Software, San Diego, CA 92,108). After surgery, HER2 expression status was evaluated by IHC in tumor pathological sections of all 34 patients. For patients with an IHC score of HER2 (2 +), FISH was further performed.

### Statistical analysis

Quantitative data were expressed as the mean ± SD. Statistical analysis of image parameters and biodistribution was performed with Student’s t test in Prism 7.0 (GraphPad Software, Inc., USA). *P* < 0.05 was considered statistically significant. Correlation analysis of the SUV data of clinical images and the IHC results was performed with SPSS Statistics 23.0 software (IBM SPSS, USA). The correlation between the SPECT T/B ratio and HER2 status was evaluated by Wilcoxon’s rank sum test. Spearman’s rank correlation analysis was performed on the SPECT T/B ratio and the immunoreactivity score. ROC curve analysis was performed to determine an optimal cutoff value for detecting HER2-positive (2 + , 3 +) breast cancer and to compare the diagnostic performance of different methods.

## Supplementary Information

**Additional file 1: Figure S1.** The synthetic route of HP-Ark2. **Figure S2.** Peptide characterization by HPLC and mass spectrometry. **Figure S3.** Metabolic stability of ^99m^Tc-H10F. **Figure S4.** Binding affinities of rk toward EGFR family proteins. **Figure S5.** NanoScan SPECT/CT imaging of ^99m^Tc-HP-Ark2 in the small tumor model. **Figure S6.** Biodistribution of ^99m^Tc-HP-Ark2 in control models. **Figure S7.** Colocalized staining of FITC-trastuzumab and Cy5-Ark2 in tumor tissues by confocal microscopy. **Figure S8.** Pharmacokinetic evaluation of ^99m^Tc-HP-Ark2 in mice. **Figure S9.** Safety evaluation of ^99m^Tc-HP-Ark2 in mice. **Figure S10.** Quantified biodistribution of ^99m^Tc-HP-Ark2 in patients. **Figure S11.** Correlation of PET T/B ratio and IHC score as well as receiver operating characteristic curve (ROC) analysis. **Figure S12.** Correlation of the T/B ratio and IHC score plus FISH results as well as receiver operating characteristic curve (ROC) analysis. **Table S1.** Estimated effective dose equivalent of ^99m^Tc-HP-Ark2 for humans.

## Data Availability

All data are available in the main text or the supplementary materials.

## Abbreviations

Ark: 8-Aminooctanoic-_D_ (RNWELRLK)
Ark2: Glu [8-aminooctanoic-_D_ (RNWELRLK)]_2_
AUC: Area under the curve
Counts_avg_: Average counts
Counts_max_: Maximum counts
DIEA: N,N-Diisopropylethylamine
DMF: N,N-Dimethylformamide
EDC: 1-(3-Dimethylaminopropyl)-3-ethylcarbodiimide hydrochloride
EGFR: Epidermal growth factor receptor
FBS: Fetal bovine serum
FISH: Fluorescence in situ hybridization
H10F: KLRLEWNR
HER2: Human epidermal growth factor receptor 2
IC: Intraductal carcinoma
IDC: Invasive ductal carcinoma
IHC: Immunohistochemistry
ILC: Invasive lobular carcinoma
KD: Dissociation constants
MeC: Medullary carcinoma
MFI: Mean fluorescence intensity
MuC: Mucinous carcinoma
NHS: N-hydroxysuccinimide
PBS: Phosphate-buffered saline
PCR: Polymerase chain reaction
PET/CT: Positron emission tomography/computed tomography
PR: Partial response
radio-HPLC: Radioactive high-performance liquid chromatography
RCP: Radiochemical purity
rk: _D_RNWELRLK
ROC curve: Receiver operating characteristic curve
SPECT/CT: Single-photon emission computed tomography/computed tomography
SPR: Surface plasmon resonance
STR: Short tandem repeat
SUV: Standardized uptake values
SUV_max_: Maximum standardized uptake value
SUV_mean_: Mean standardized uptake value
T/B ratio: Ratio of tumor-to-background (breast)
^99m^Tc-HP-Ark2: ^99m^Tc-{HYNIC-PEG_4_-Glu [8-aminooctanoic-_D_(RNWELRLK)]_2_/Tricine/TPPTS}
TFA: Trifluoroacetic acid
TPPTS: Trisodium triphenylphosphine-3,3',3''-trisulfonate
Tricine: N-[Tris (hydroxymethyl) methyl] glycine
VOI: Volume of interest
