# Supplementary material for: Molecular imaging of HER2 expression in breast cancer patients using a novel peptide-based tracer 99mTc-HP-Ark2: a pilot study
Source: J Transl Med. 2023 Jan 11;21:19. doi: 10.1186/s12967-022-03865-y (PMC9835228; doi:10.1186/s12967-022-03865-y)
Supplement: Supplementary file 1 — Additional file 1: Figure S1. The synthetic route of HP-Ark2. Figure S2. Peptide characterization by HPLC and mass spectrometry. Figure S3. Metabolic stability of 99mTc-H10F. Figure S4. Binding affinities of rk toward EGFR family proteins. Figure S5. NanoScan SPECT/CT imaging of 99mTc-HP-Ark2 in the small tumor model. Figure S6. Biodistribution of 99mTc-HP-Ark2 in control models. Figure S7. Colocalized staining of FITC-trastuzumab and Cy5-Ark2 in tumor tissues by confocal microscopy. Figure S8. Pharmacokinetic evaluation of 99mTc-HP-Ark2 in mice. Figure S9. Safety evaluation of 99mTc-HP-Ark2 in mice. Figure S10. Quantified biodistribution of 99mTc-HP-Ark2 in patients. Figure S11. Correlation of PET T/B ratio and IHC score as well as receiver operating characteristic curve (ROC) analysis. Figure S12. Correlation of the T/B ratio and IHC score plus FISH results as well as receiver operating characteristic curve (ROC) analysis. Table S1. Estimated effective dose equivalent of 99mTc-HP-Ark2 for humans. [file 12967_2022_3865_MOESM1_ESM.doc]

**Supplementary Materials**

Methods for pharmacokinetic and safety evaluation of ^99m^Tc-HP-Ark2; preliminary evaluation of trastuzumab treatment in a patient by ^99m^Tc-HP-Ark2 SPECT/CT.

Figure S1. The synthetic route of HP-Ark2.

Figure S2. Peptide characterization by HPLC and mass spectrometry.

Figure S3. Metabolic stability of ^99m^Tc-H10F.

Figure S4. Binding affinities of rk toward EGFR family proteins.

Figure S5. NanoScan SPECT/CT imaging of ^99m^Tc-HP-Ark2 in the small tumor model.

Figure S6. Biodistribution of ^99m^Tc-HP-Ark2 in control models.

Figure S7. Colocalized staining of FITC-trastuzumab and Cy5-Ark2 in tumor tissues by confocal microscopy.

Figure S8. Pharmacokinetic evaluation of ^99m^Tc-HP-Ark2 in mice.

Figure S9. Safety evaluation of ^99m^Tc-HP-Ark2 in mice.

Figure S10. Quantified biodistribution of ^99m^Tc-HP-Ark2 in patients.

Figure S11. Correlation of the T/B ratio and IHC score plus FISH results as well as receiver operating characteristic curve (ROC) analysis.

Figure S12. Correlation of the T/B ratio and IHC score plus FISH results as well as receiver operating characteristic curve (ROC) analysis.

Table S1. Estimated effective dose equivalent of ^99m^Tc-HP-Ark2 for humans.

**Pharmacokinetic and safety evaluation of ^99m^Tc-HP-Ark2**

Seven 4-week-old female ICR mice were used to evaluate the blood clearance of ^99m^Tc-HP-Ark2. Each mouse was injected with 1.85 MBq/50 μCi of ^99m^Tc-HP-Ark2 via the tail vein. Blood was drawn through the orbit of the mouse at 1, 3, 8, 15, 30, 60, 90, 120, 180, and 240 min p.i. The radioactivity was counted in a γ-counter, and the percentage injected dose per gram of blood (%ID/g) at each time point was calculated. The slow half-life and fast half-life were analyzed and calculated by Prism 7.0.

Ten 4-week-old female ICR mice were randomly divided into the experimental and control groups. The mice were numbered, weighed and photographed. The mice in the experimental group were injected with 74 MBq/2 mCi (500 μL) of ^99m^Tc-HP-Ark2 via the tail vein, and the injection was completed within 5 seconds. The mice in the control group were injected with the same volume of saline. After 120 h, the mice were photographed again, their body weight was recorded, and the behavioral differences were observed. After the mice were sacrificed, they were dissected to observe the morphological differences in the organs.

The blood clearance curve of ^99m^Tc-HP-Ark2 is shown in **Figure S8**. The slow half-life and fast half-life of ^99m^Tc-HP-Ark2 in blood were measured as 32.45 and 3.12 min, respectively. As shown in **Figure S9**, the body weight of ICR mice did not decrease after high-dose injection of ^99m^Tc-HP-Ark2 (74 MBq) but rather increased steadily, as in the control group. Moreover, no abnormal behavior was observed in mice 120 h after injection, and no abnormal organs were observed after dissection.

The radiation effective dose (mSv/MBq) of the main organs of the human body was estimated from the biodistribution data of the SK-BR-3 tumor model (**Table S1**). The calculated mean effective dose equivalent for the whole body was 9.17 ×10^-3^ mSv/MBq for males and 1.03 ×10^-2^ mSv/MBq for females. The mean injected activity was 11.1 MBq/kg, the effective radiation dose of ^99m^Tc-HP-Ark2 to the body was approximately 6.62 mSv and 5.72 mSv for 65 kg males and 50 kg females, respectively, which were in the same range of the estimated effective dose as the clinically widely used ^18^F-FDG (7.40 mSv for males and 9.25 mSv for females) [1, 2], indicating the safety of its application *in vivo*.


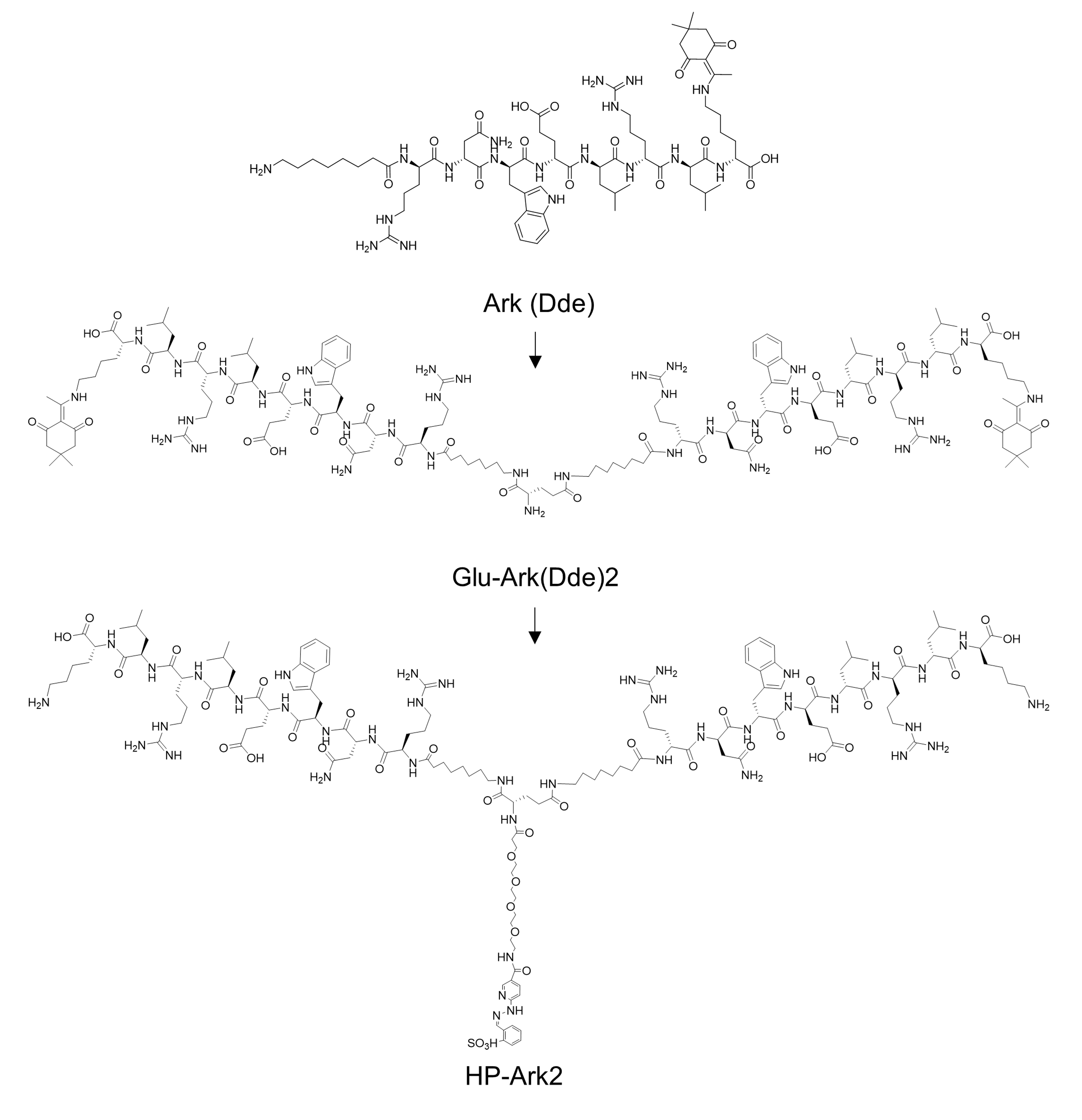


**Figure S1. The synthetic route of HYNIC-PEG_4_-Ark2 (HP-Ark2).**


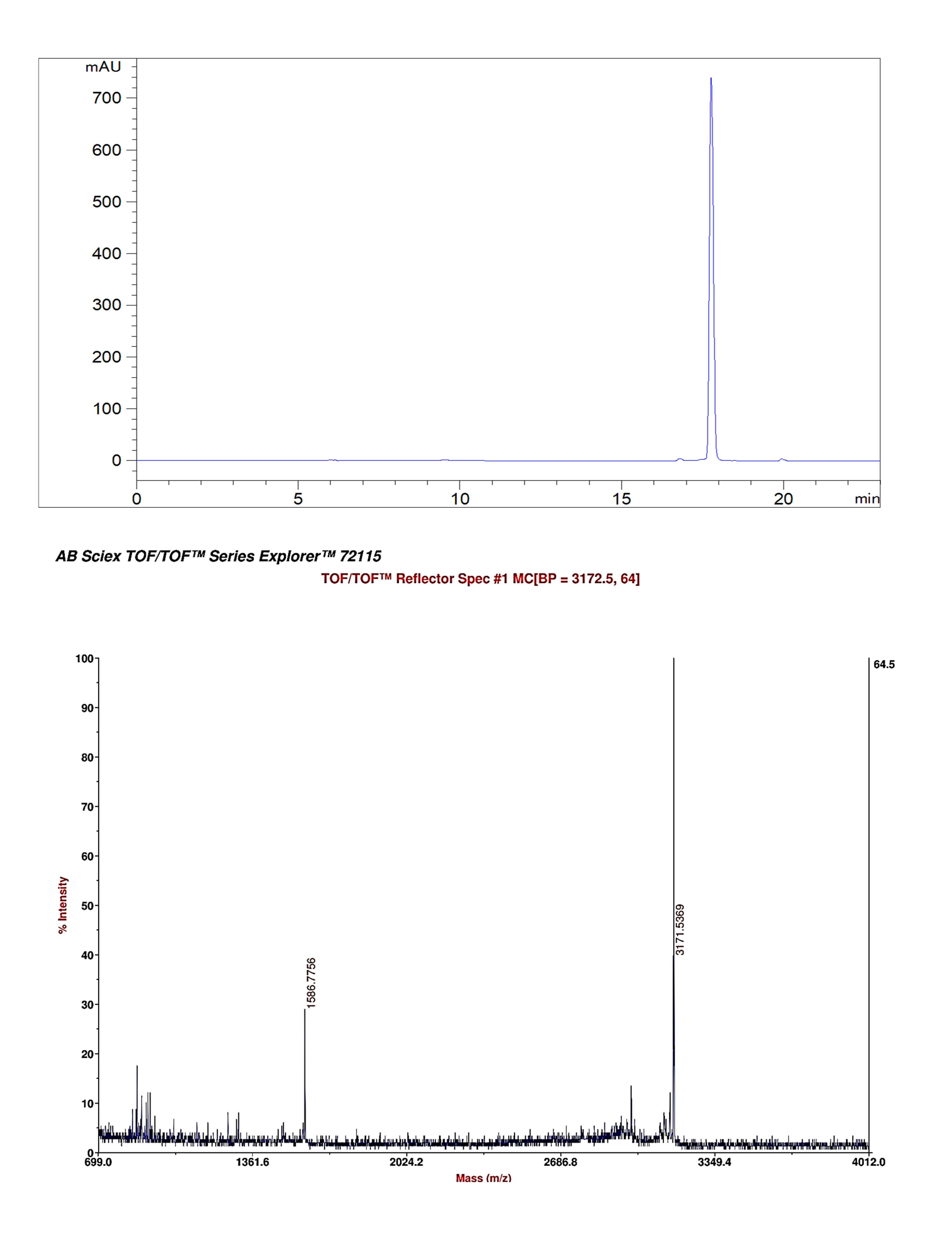


**Figure S2. Peptide characterization by HPLC and mass spectrometry.** Typical HPLC chromatogram (top) and MALDI-TOF mass spectrometry of HP-Ark2 (bottom). The experiments were replicated 3 times.


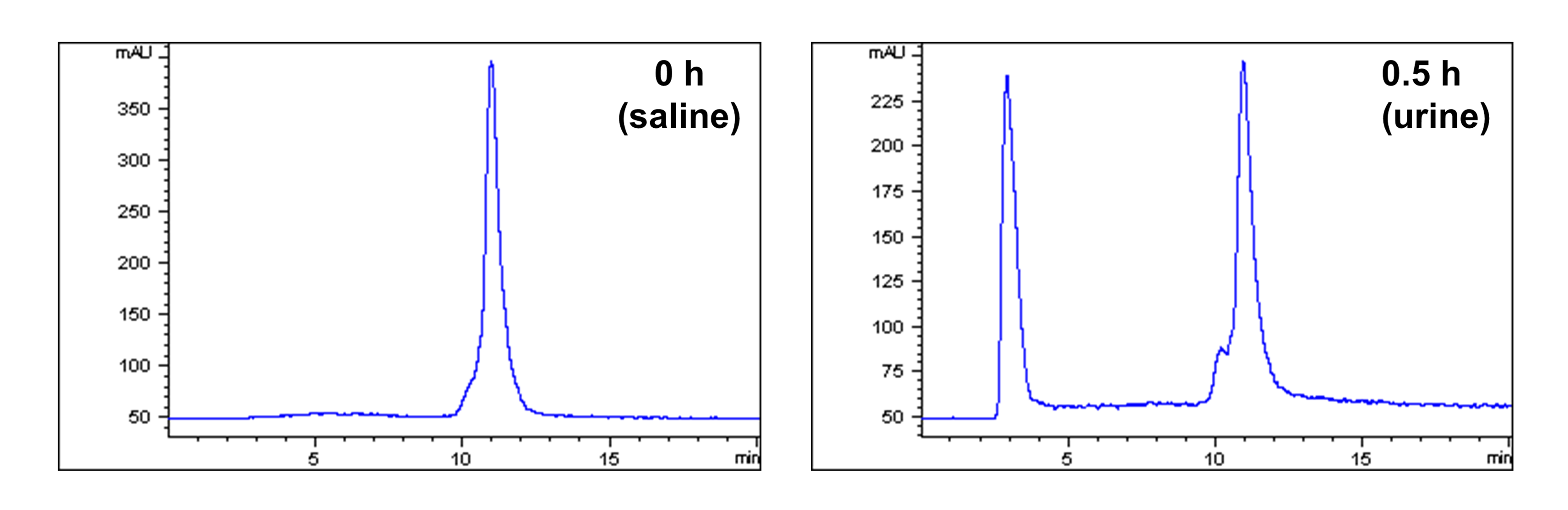


**Figure S3. Metabolic stability of ^99m^Tc-H10F.** Radiochemical purity of ^99m^Tc-H10F at 0 h (in saline) and *in vivo* metabolic stability at 0.5 h (in urine). The experiment was replicated twice.


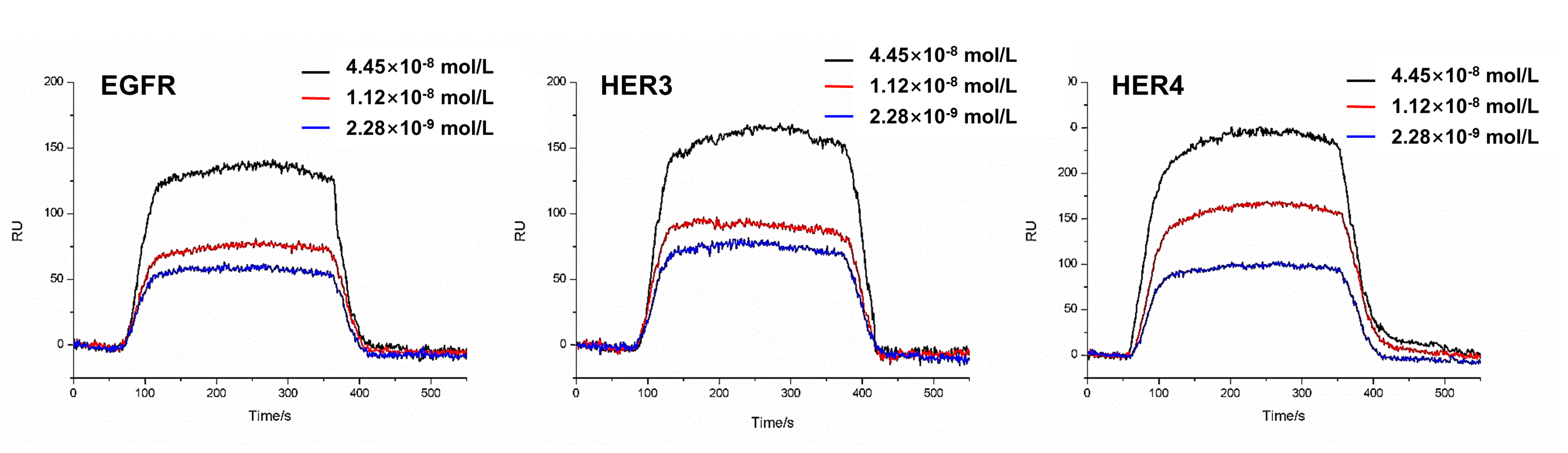


**Figure S4.** **Binding affinities of rk toward EGFR family proteins.** SPR detection of the rk peptide binding affinities toward EGFR, HER3 and HER4 proteins. The experiment was replicated twice.


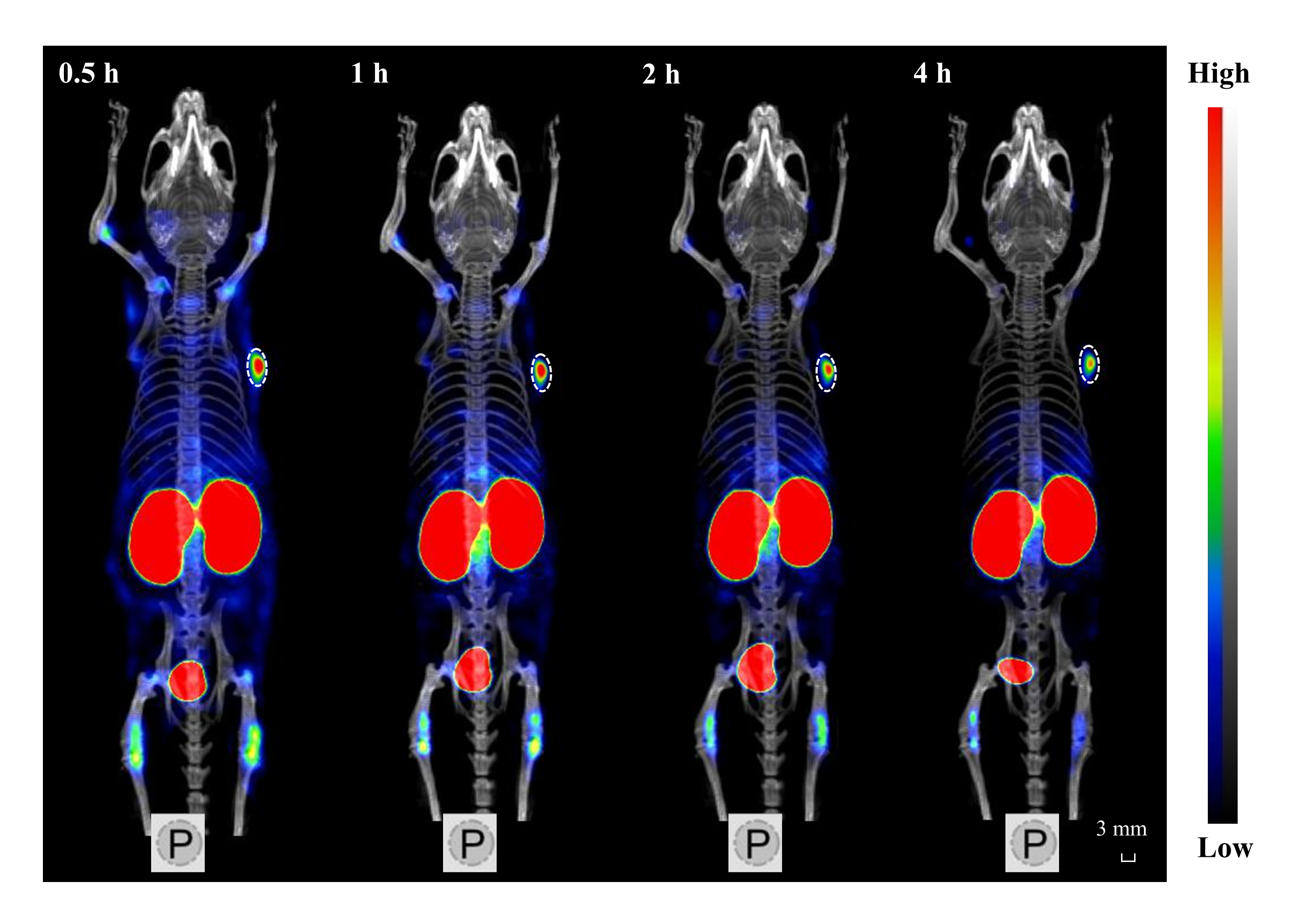


**Figure S5.** **NanoScan SPECT/CT imaging of ^99m^Tc-HP-Ark2 in the small tumor model.** NanoScan SPECT/CT imaging of ^99m^Tc-HP-Ark2 in the SK-BR-3 model of small tumors (~20 mm^3^) at 0.5, 1, 2 and 4 h postinjection (n = 3). The experiment was replicated 3 times.


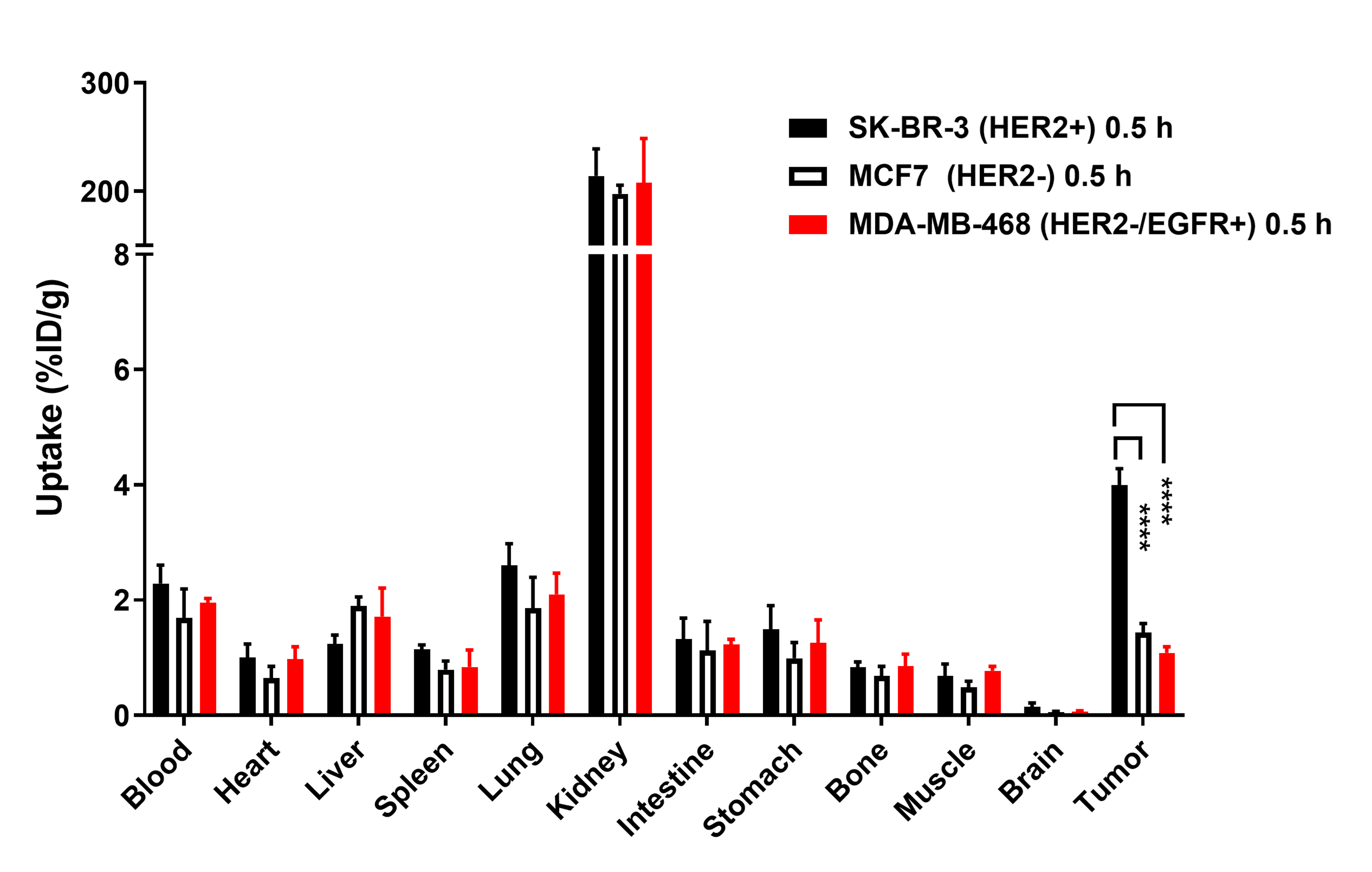


**Figure S6. Biodistribution of ^99m^Tc-HP-Ark2 in control models.** Biodistribution of ^99m^Tc-HP-Ark2 in the HER2-positive SK-BR-3 tumor model as well as the HER2-negative MCF7 and MDA-MB-468 tumor models at 0.5 h postinjection (n = 4). The experiment was replicated twice.


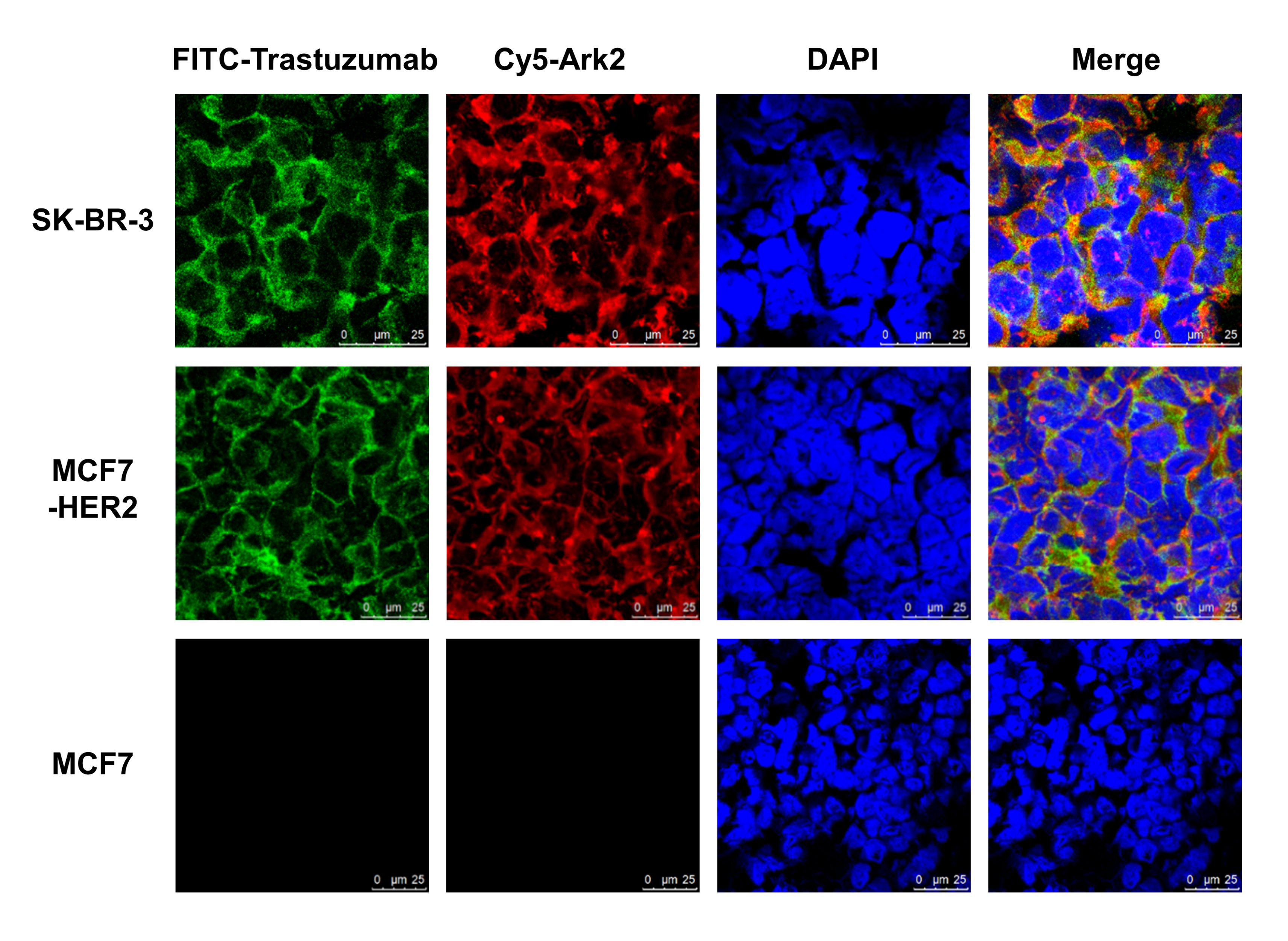


**Figure S7. Colocalized staining of FITC-trastuzumab and Cy5-Ark2 in tumor tissues by confocal microscopy.** Confocal images of FITC-trastuzumab (green) and Cy5-Ark2 (red) staining of SK-BR-3 (HER2-positive), MCF7-HER2 (HER2-positive) and MCF7 (HER2-negative) tumor tissues. Colocalized staining of FITC-trastuzumab and Cy5-Ark2 in tumor tissues is shown in the merged images. The experiments were replicated twice.


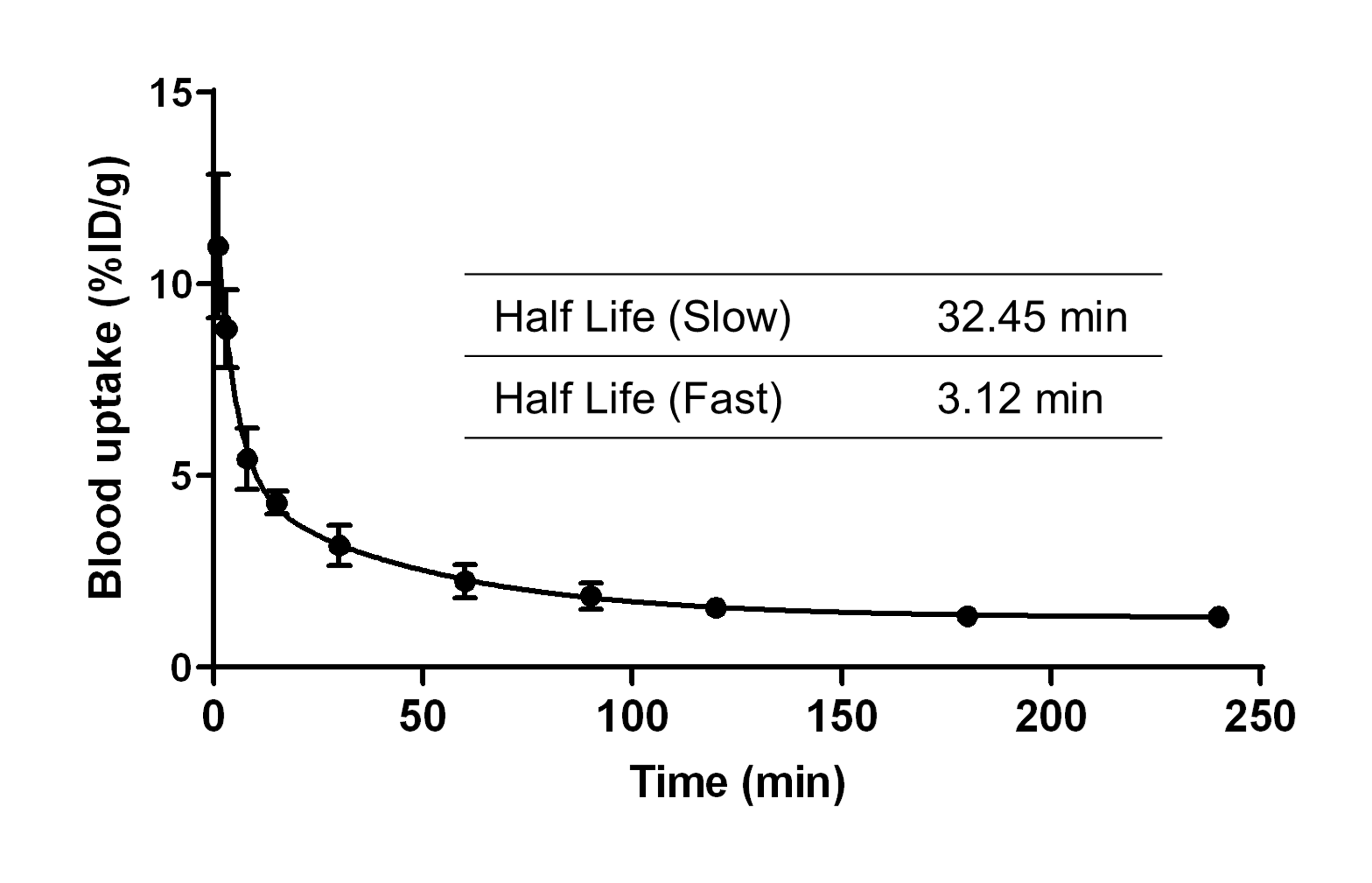


**Figure S8. Pharmacokinetic evaluation of ^99m^Tc-HP-Ark2 in mice.** Blood clearance curve of ^99m^Tc-HP-Ark2 in ICR normal mice


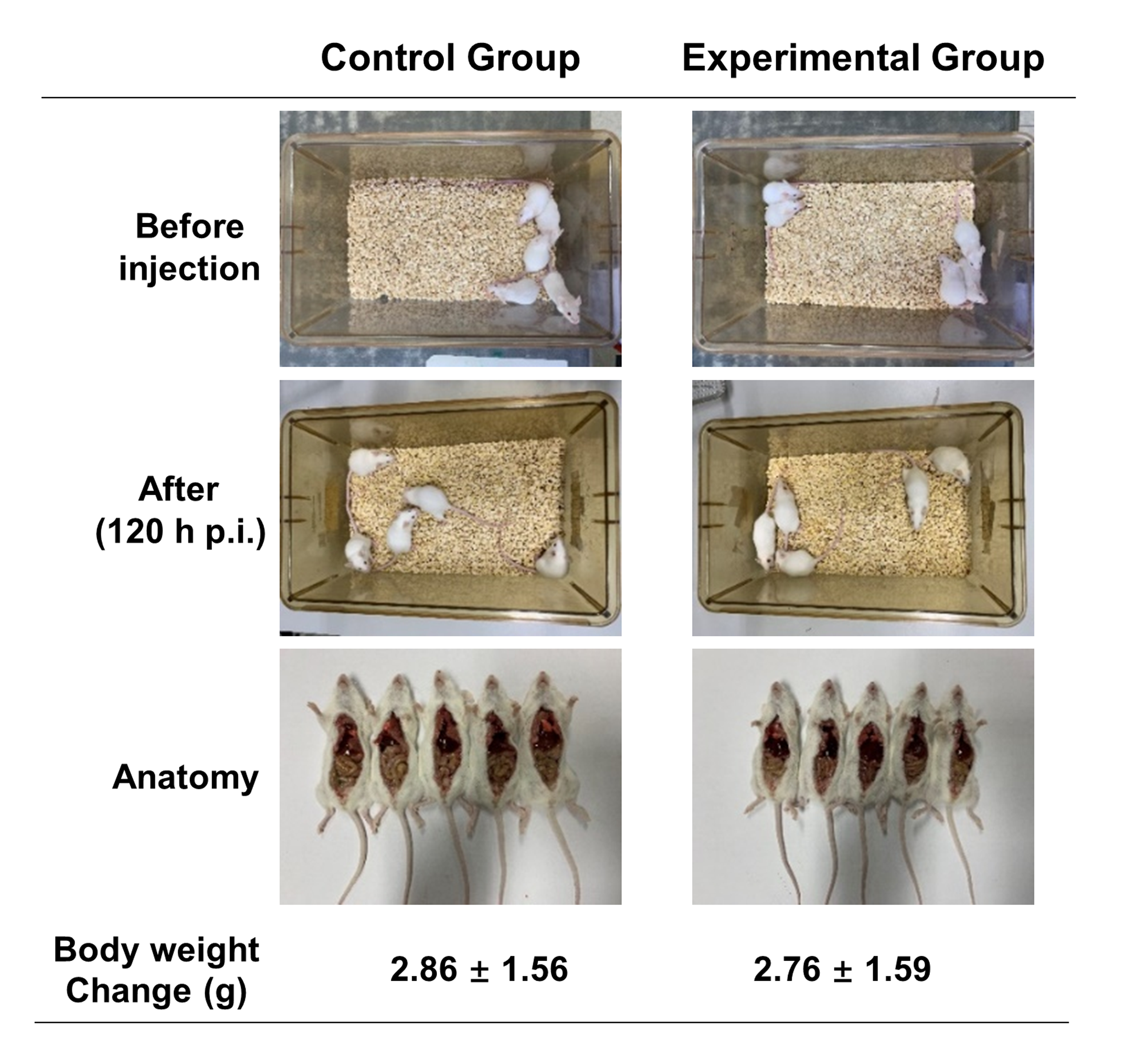


**Figure S9. Safety evaluation of ^99m^Tc-HP-Ark2 in mice.** Differences in body weight, behavior and organ morphology between the control and experimental groups after 120 h p.i. (n = 5)


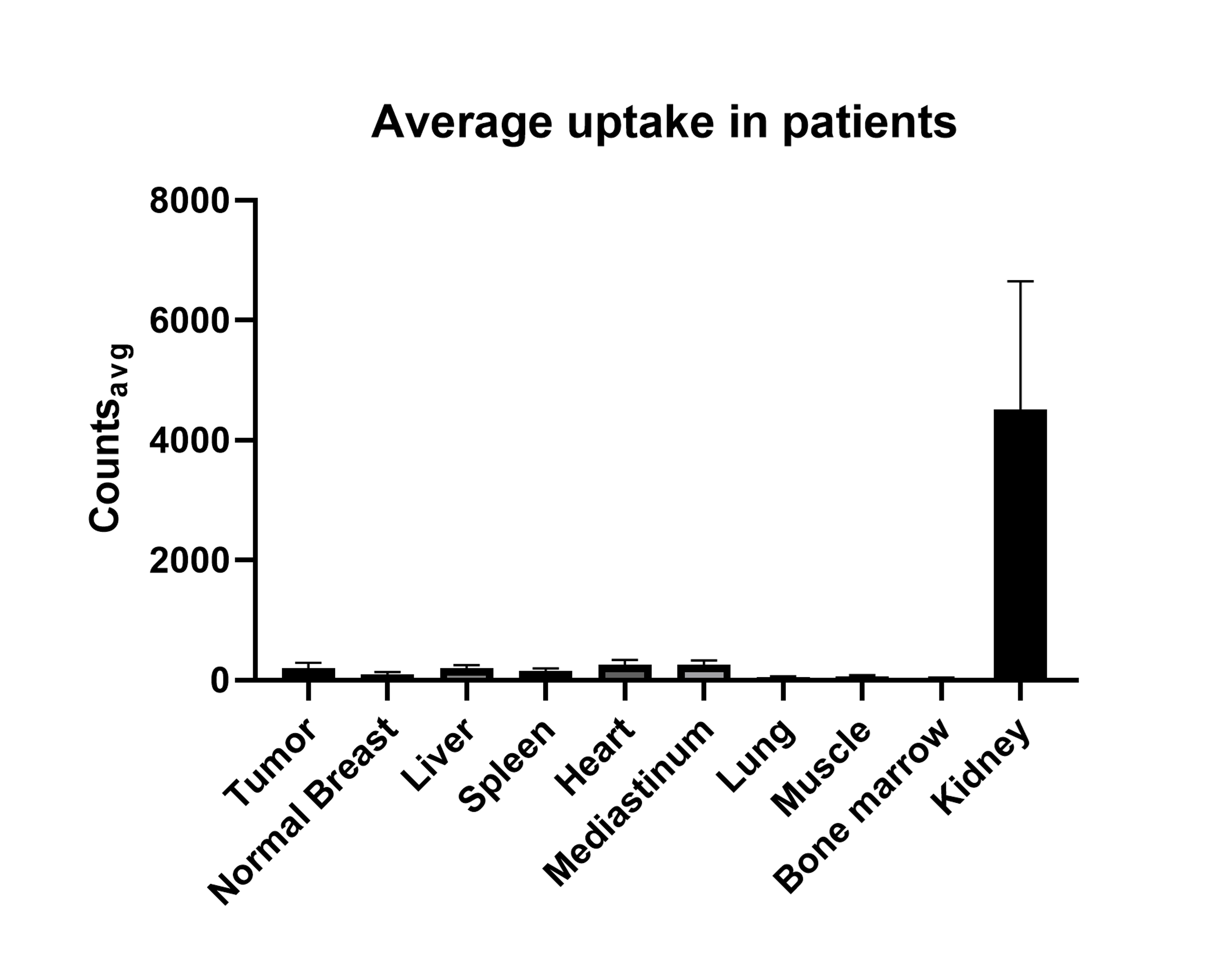


**Figure S10. Quantified biodistribution of ^99m^Tc-HP-Ark2 in patients.** Quantified biodistribution (counts) of ^99m^Tc-HP-Ark2 in breast cancer patients at 1 h postinjection, as calculated from SPECT/CT images (n = 34).


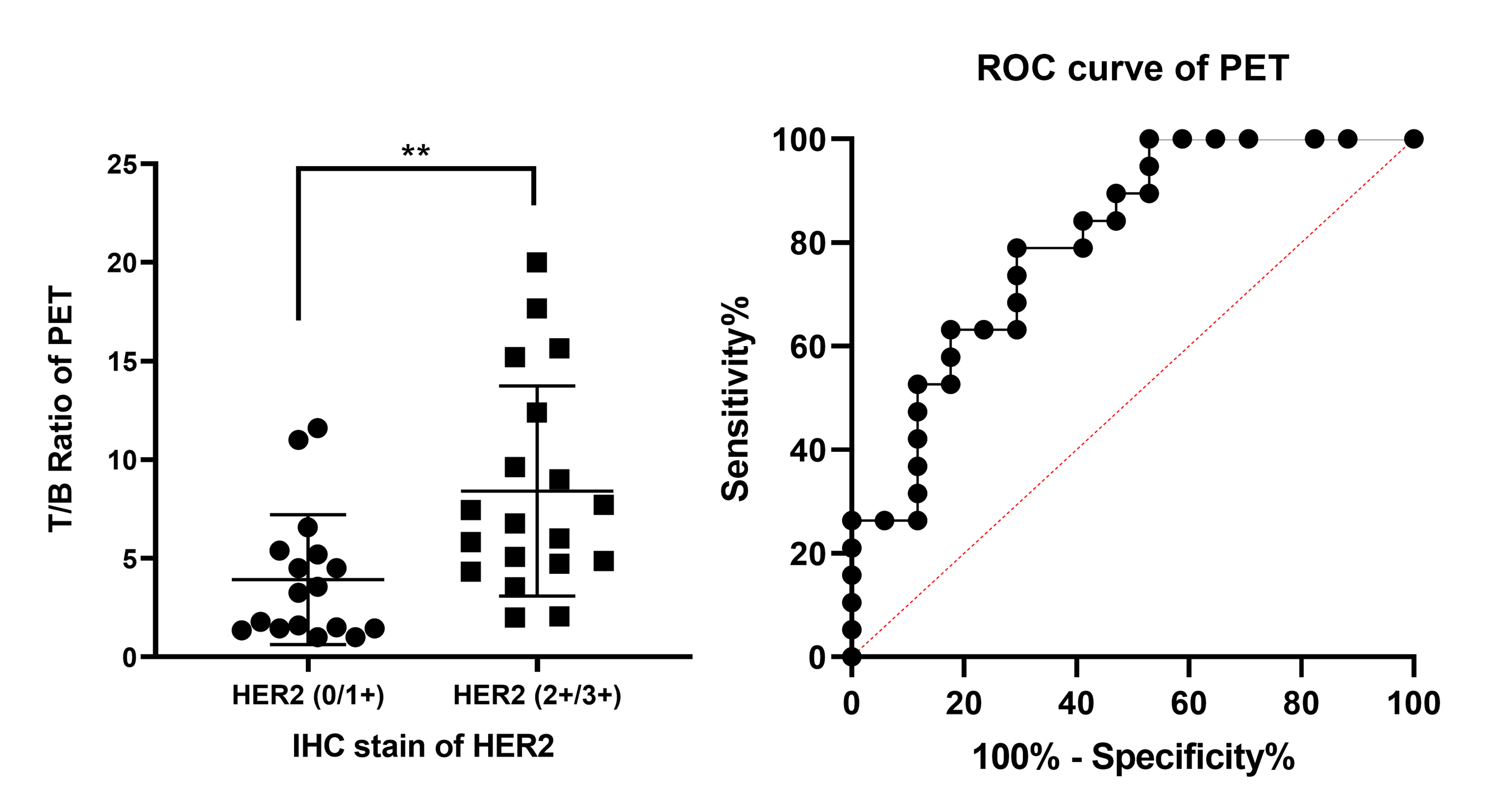


**Figure S11. Correlation of the PET T/B ratio and IHC score results as well as receiver operating characteristic curve (ROC) analysis.** The correlation of the ^18^F-FDG PET tumor T/B ratio and the HER2 status (IHC) of breast cancer (A). The ROC analysis of ^18^F-FDG PET in HER2-positive (2+, 3+) breast lesions (B) (n = 36).


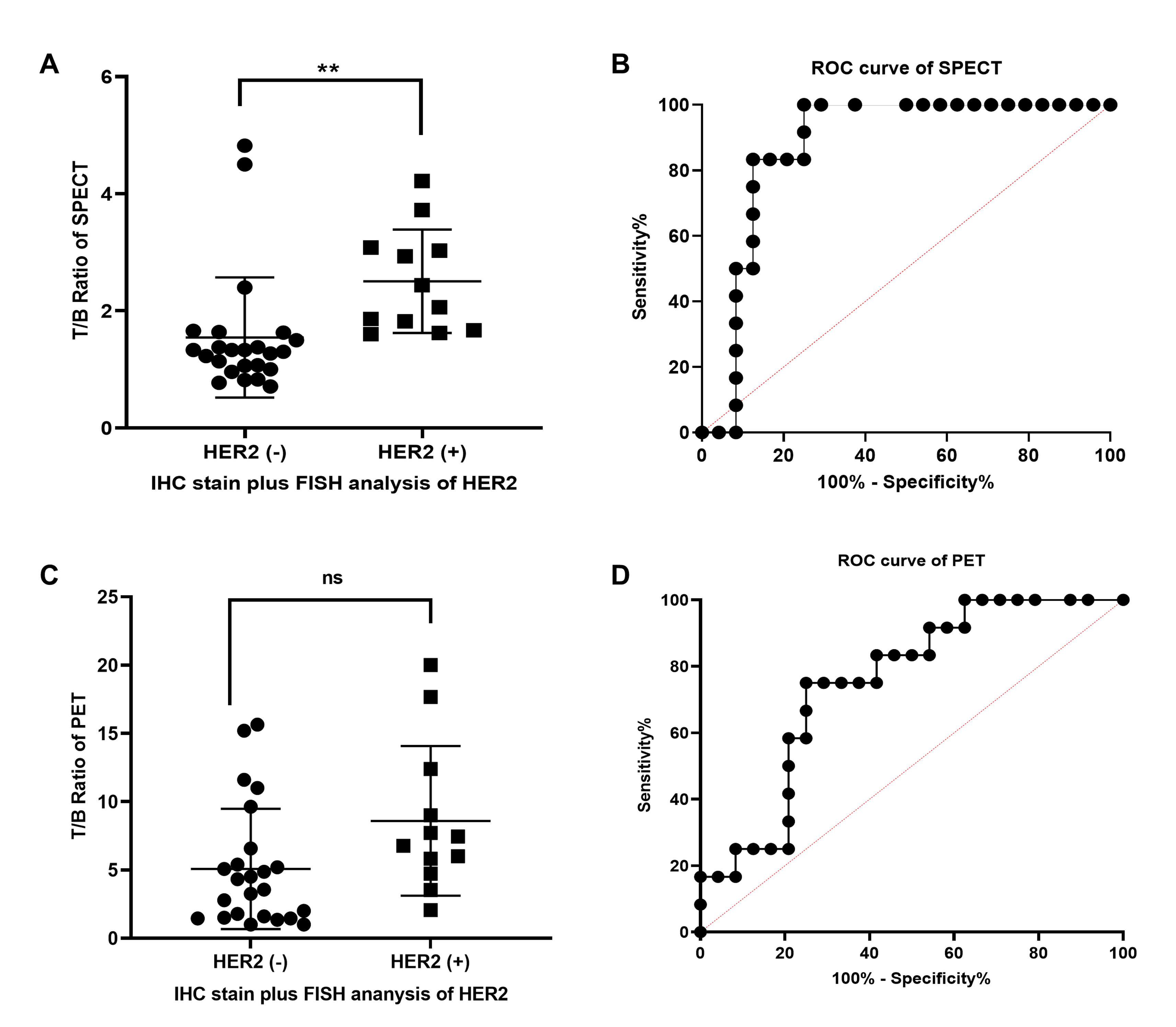


**Figure S12. Correlation of the T/B ratio and IHC score plus FISH results as well as** **the receiver operating characteristic curve (ROC) analysis.** The correlation of the ^99m^Tc-HP-Ark2 SPECT tumor T/B ratio and the HER2 status (IHC+FISH) of breast cancer (A). The ROC analysis of ^99m^Tc-HP-Ark2 SPECT in HER2-positive (2+ plus FISH+, 3+) breast lesions (B). The correlation of the ^18^F-FDG PET tumor T/B ratio and the HER2 status (IHC+FISH) of breast cancer (C). The ROC analysis of ^18^F-FDG PET in HER2-positive (2+ plus FISH+, 3+) breast lesions (D) (n = 36).

**Table S1. Estimated effective dose equivalent of ^99m^**Tc-HP-Ark2 for humans.

| **Effective Dose Equivalent (mSv/MBq)** | | |
| --- | --- | --- |
| **Target Organ** | **Male** | **Female** |
| **Adrenals** | 4.04E-04 | 5.36E-04 |
| **Brain** | 0.00E+00 | 0.00E+00 |
| **Breasts** | 3.64E-05 | 4.44E-05 |
| **Gallbladder Wall** | 2.31E-04 | 2.60E-04 |
| **LLI Wall** | 0.00E+00 | 0.00E+00 |
| **Small Intestine** | 0.00E+00 | 0.00E+00 |
| **Stomach Wall** | 0.00E+00 | 0.00E+00 |
| **ULI Wall** | 0.00E+00 | 0.00E+00 |
| **Heart Wall** | 0.00E+00 | 0.00E+00 |
| **Kidneys** | 7.26E-03 | 7.87E-03 |
| **Liver** | 0.00E+00 | 0.00E+00 |
| **Lungs** | 1.38E-04 | 1.90E-04 |
| **Muscle** | 0.00E+00 | 0.00E+00 |
| **Ovaries** | / | 2.49E-04 |
| **Pancreas** | 2.81E-04 | 3.43E-04 |
| **Red Marrow** | 1.94E-04 | 2.27E-04 |
| **Osteogenic Cells** | 4.73E-05 | 6.07E-05 |
| **Skin** | 0.00E+00 | 0.00E+00 |
| **Spleen** | 3.96E-04 | 4.89E-04 |
| **Testes** | 0.00E+00 | / |
| **Thymus** | 0.00E+00 | 0.00E+00 |
| **Thyroid** | 2.27E-06 | 2.78E-06 |
| **Urinary Bladder Wall** | 0.00E+00 | 0.00E+00 |
| **Uterus** | 0.00E+00 | 0.00E+00 |
| **Total Body** | 9.17E-03 | 1.03E-02 |

References

1. Verbruggen A, Coenen HH, Deverre JR, Guilloteau D, Langstrom B, Salvadori PA, et al. Guideline to regulations for radiopharmaceuticals in early phase clinical trials in the EU. Eur J Nucl Med Mol Imaging. 2008; 35: 2144-51.

2. Kaushik A, Jaimini A, Tripathi M, D'Souza M, Sharma R, Mondal A, et al. Estimation of radiation dose to patients from (18) FDG whole body PET/CT investigations using dynamic PET scan protocol. Indian J Med Res. 2015; 142: 721-31.
